# Intake of dairy products and associations with major atherosclerotic cardiovascular diseases: a systematic review and meta-analysis of cohort studies

**DOI:** 10.1038/s41598-020-79708-x

**Published:** 2021-01-14

**Authors:** Marianne Uhre Jakobsen, Ellen Trolle, Malene Outzen, Heddie Mejborn, Manja G. Grønberg, Christian Bøge Lyndgaard, Anders Stockmarr, Stine K. Venø, Anette Bysted

**Affiliations:** 1 Division for Diet, Disease Prevention and Toxicology, National Food Institute, Technical University of Denmark, Kgs. Lyngby, Denmark; 2 Division for Risk Assessment and Nutrition, National Food Institute, Technical University of Denmark, Kgs. Lyngby, Denmark; 3 Department of Applied Mathematics and Computer Science, Technical University of Denmark, Kgs. Lyngby, Denmark; 4 Department of Cardiology, Aalborg University Hospital, Aalborg, Denmark; 5 Division for Food Technology, National Food Institute, Technical University of Denmark, Kgs. Lyngby, Denmark

**Keywords:** Disease prevention, Nutrition, Public health, Cardiovascular diseases

## Abstract

Specific types of dairy products may be differentially associated with atherosclerotic cardiovascular disease (CVD). We conducted a systematic review and meta-analysis of cohort studies to summarize findings on the associations between total dairy product intake and intake of dairy product subgroups and the risk of major atherosclerotic CVDs in the general adult population. Our protocol was registered in PROSPERO (CRD42019125455). PubMed and Embase were systematically searched through 15 August 2019. For high versus low intake and dose–response meta-analysis, random-effects modelling was used to calculate summary risk ratios (RR). There were 13 cohort studies included for coronary heart disease (CHD), 7 for ischemic stroke and none for peripheral artery disease. High-fat milk was positively associated with CHD (RR 1.08 (95% confidence interval 1.00–1.16) per 200 g higher intake/day) and cheese was inversely associated with CHD (RR 0.96 (95% confidence interval 0.93–0.98) per 20 g higher intake/day). Heterogeneity, however, was observed in high versus low meta-analyses. Milk was inversely associated with ischemic stroke in high versus low meta-analysis only. In conclusion, this systematic review indicates a positive association of high-fat milk and an inverse association of cheese with CHD risk. The findings should be interpreted in the context of the observed heterogeneity.

## Introduction

Elevated low-density lipoprotein (LDL)-cholesterol is an important risk factor of atherosclerosis (a disease of the large arteries), which is the primary cause of atherosclerotic cardiovascular diseases (CVDs)^1^. Nutrient trials have shown that intake of saturated fatty acids increases serum LDL-cholesterol relative to intake of unsaturated fatty acids and carbohydrates^2^. As whole-fat dairy products have a high saturated fat content, food-based dietary guidelines recommend consumption of low-fat dairy products in place of high-fat dairy products^3^. However, the health effects of saturated fat varies depending on the specific fatty acid and possibly by the specific food source^4,5^.

The role of dairy foods in CVD prevention has been reviewed and summarized^6–9^. Because dairy is a heterogeneous food group of solid, semi solid and liquid fermented or non-fermented products, differing in nutrients and probiotics found in fermented dairy products, the focus has been on the intake of specific types of dairy products in relation to CVD development rather than on total intake of dairy products. In a systematic review and meta-analysis from 2017 by Guo et al.^9^ associations between intake of milk, yogurt and cheese and risk of coronary heart disease (CHD) were summarized. No associations between intake of any of the investigated dairy product subgroups and CHD were observed. In a systematic review and meta-analysis from 2016 by de Goede et al.^6^ associations between intake of milk, yogurt, cheese and butter and risk of stroke were summarized. A higher intake of milk was found associated with a lower risk of stroke, and a higher intake of high-fat milk was found to be associated with a higher risk of stroke. No associations between the intake of low-fat milk, yogurt, cheese or butter and stroke were observed. In subgroup analyses by stroke type (ischemic and hemorrhagic), no associations were observed with milk intake. Associations between stroke type and intake of low-fat milk or high-fat milk were not considered; nor were associations with other dairy product subgroups.

We conducted a systematic review and meta-analysis of cohort studies to summarize the findings on the associations between total intake of dairy products and intake of dairy product subgroups (milk, yogurt, cheese and butter) and the risk of major atherosclerotic CVDs (total (the sum of fatal and nonfatal) CHD, total ischemic stroke and peripheral artery disease) in the general adult population. Data synthesis was also differentiated according to the fat content of the dairy product subgroups. The focus of this systematic review is on *atherosclerotic* CVDs because dairy products may play an important role in prevention of atherosclerotic CVDs. We included only studies considering total CHD and total ischemic stroke as outcomes. Studies on total CHD and total ischemic stroke may be seen as studies on etiology of CHD and ischemic stroke, respectively, whereas studies on only fatal outcomes may be seen as studies exploring combined etiology and prognosis. The associations between dairy and major atherosclerotic CVDs were investigated using high versus low intake and dose–response (linear and non-linear) meta-analysis.

## Methods

### Protocol and registration

This systematic review and meta-analysis was planned and conducted according to the preferred reporting items for systematic reviews and meta-analyses (PRISMA)^10,11^. The systematic review protocol, including review question, search strategy, eligibility criteria (participants, exposure, comparator, outcome and study design (PECOS) items) and risk of bias assessment, was registered in PROSPERO International Prospective Register of Systematic Reviews (http://www.crd.york.ac.uk/PROSPERO, identifier CRD42019125455) ahead of conducting the review. The PRISMA checklist has been submitted to the journal as an attachment to this article (see Supplementary Table S1 online).

### Eligibility criteria

*Participants:* Participants recruited from the general adult population.

*Exposure:* Considering at least one of the exposures total intake of dairy products, intake of low-fat dairy products, intake of high-fat dairy products, intake of milk, intake of low-fat milk (fat content lower than whole-fat milk), intake of high-fat milk (fat content equalling whole-fat milk), intake of yogurt/other soured milk products (referred to hereinafter as yogurt), intake of low-fat yogurt/other soured milk products (fat content lower than whole-fat yogurt/other soured milk products, referred to hereinafter as low-fat yogurt), intake of high-fat yogurt/other soured milk products (fat content equalling whole-fat yogurt/other soured milk products, referred to hereinafter as high-fat yogurt), intake of cheese, intake of low-fat cheese, intake of high-fat cheese and intake of butter.

*Comparator:* When the exposure is total intake of dairy products, the comparator is a lower intake of total intake of dairy products and when the exposure is intake of a dairy product subgroup, the comparator is a lower intake of that particular dairy product subgroup.

*Outcome:* Considering at least one of the outcomes total CHD, total ischemic stroke and peripheral artery disease.

*Study design:* Cohort study that provides evidence about associations between exposures and incidence of hard outcomes.

Only published articles were considered. There were no restrictions to language.

### Search

Studies were identified by searching the bibliographic databases PubMed and Embase (through 15 August 2019). The search included only terms related to exposures and outcomes. The full literature search strategy for PubMed is shown in Supplementary Methods online. Additionally, we checked the reference lists of the included studies. Previous reviews and meta-analyses were also used as an information source.

### Study selection

An eligibility assessment was performed independently in a standardised manner by two reviewers (M.U.J. and M.O. or M.U.J. and E.T.). Titles and/or abstracts of records identified through the search were screened against the eligibility criteria. The full text of articles that appeared to meet the study eligibility criteria, or where there was any uncertainty about eligibility, was retrieved and assessed for eligibility. Any disagreement between the two reviewers over the eligibility assessment of a particular study was resolved by discussion. The PRISMA flow diagram^11^ was used to summarize the study selection processes.

### Data collection process and data items

A data extraction sheet (developed a priori by the reviewers and pilot-tested) was used to extract data from the included studies. One reviewer (M.U.J.) extracted the following data items from the studies and a second reviewer (M.O., E.T., M.G.G., C.B.L. or S.K.V.) checked the extracted data: First author’s last name, publication year, cohort name, study origin, recruitment year/period, gender, age at entry, sample size, exclusion criteria, exposure and exposure definition, exposure frequency and quantity (M.O.); method for collecting information on exposure (E.T.); outcome, method for ascertainment of outcome (S.K.V.); length of follow-up, lost to follow-up, adjustment variables; total number of events (M.O.); number of events per exposure level, number of participants or person-years per exposure level (M.G.G. or C.B.L.); point risk estimates for associations and their confidence limits, funding sources (M.O.). When risk estimates from more than one multivariable analysis were reported, we extracted data from the analysis adjusting for the largest number of confounders. Moreover, if risk estimates from specified substitution analyses were reported, only risk estimates for substitutions between dairy products were extracted. Any disagreement between the two reviewers over the extracted data of a particular study was resolved by discussion. We contacted (by e-mail) 12 authors^12–23^ of the included studies for further information on study methods (exposure definition, quantitative amount) and results (number of events per exposure category, number of participants or person-years per exposure category). In case a study was published more than once, we included the most comprehensive study in terms of exposures considered or sample size if exposure was the same.

### Risk of bias in individual studies

Risk of bias judgements of the included studies were based on the Newcastle–Ottawa scale (NOS) tool^24^. Two reviewers (M.U.J. and E.T. or M.U.J. and S.K.V.) independently assessed the internal validity of the included studies by assessing risk of selection bias, information bias and confounding. Any disagreement between the two reviewers over the risk of bias judgements (“low risk” of bias, “high risk” of bias, “unclear risk” of bias) of a particular study was resolved by discussion. A cross-tabulation of study by quality item was used to present the risk of bias judgements. A study was classified as being at low risk of bias in general only if ≤ 2 of the 8 items established a “high risk” or “unclear risk” of bias.

### Data processing and statistical analysis

A summary table was used to present characteristics of the included studies. The measure of associations in the included studies were hazard ratios (the most common measure) and odds ratios, but both measures were treated as risk ratios (RRs) in the meta-analysis^25^. Standard error estimates for point risk estimates were recovered from confidence limits. Studies for which information on point risk estimates and confidence limits was not reported were left out. Where results were reported in all participants and in men and women separately, we used results in men and results in women.

When intake by exposure category was provided as a point estimate, we used this for the corresponding risk estimate. Where a range of intake was provided, we calculated the midpoint. Where a lower intake range was open-ended, we calculated the midpoint between zero and the given lower boundary; and when an upper intake range was open-ended, we assumed that its width was the same as the adjacent category. In case this category was a point estimate, then the nearest intake range was used. For studies where intake was provided only as serving size (and where the quantitative amount was not specified), we used conversion standards (see Supplementary Table S2 online).

Quantitative data synthesis was conducted where studies had used similar exposure with similar outcome. For high versus low intake and dose–response meta-analysis, a random-effects model was used to calculate summary RR estimates and corresponding 95% confidence intervals (CIs), which incorporated both within- and between-study variability^26^. The inverse variance method was used for weighting the studies and the DerSimonian & Laird estimator was used to estimate the between study variance^26^. In high versus low intake meta-analysis, the reference category was the lowest intake category (for most studies) or no intake as reported in the included studies. Linear dose–response meta-analysis was carried out as a two-stage procedure. In the first stage, a slope (trend) for each study was calculated, and in the second stage, the study-specific slope estimates were combined using the random-effects model^26^. For studies where a linear dose–response trend was reported, we used this for the analysis. For studies not reporting a linear dose–response trend, we calculated study-specific linear trends and corresponding CIs from the natural logs of the point risk estimates of the exposure categories and their confidence limits. The covariance between the log risk estimates within each study was approximated using the method of Greenland & Longnecker^27^. This method requires information on the distribution of cases and the number of participants or person-years for each exposure category. Thus, studies for which this information was not provided were left out. However, in case the distribution of participants was not provided in studies where the exposure was defined in quantiles, the distribution was calculated by dividing the total number of participants by the number of quantiles. For studies with only two exposure categories, the covariance matrix between the log risk estimates only consisted of the variance of the log risk estimate for the non-referent exposure category. Thus, no covariance matrix had to be approximated by the method of Greenland & Longnecker^27^, resulting in the exception that when only one non-referent intake category was present, the study was not left out even though the number of participants and the number of cases for the exposure categories were not provided. For linear dose–response meta-analysis, the combined trend was reported as risk per higher intake in grams per day as follows: for milk 200 g/day, yogurt 100 g/day, cheese 20 g/day, butter 6 g/day. For linear dose–response meta-analysis of substitutions between dairy product subgroups, however, the combined trend was reported as risk per 1 serving/day substituted. Studies were left out when information on quantitative amount of exposure categories was not provided. Non-linear dose–response meta-analysis was also carried out as a two-stage procedure. In the first stage, a restricted cubic spline model using three knots at 10%, 50% and 90% of the total distribution of the reported intake was applied for each study with at least three exposure categories, and in the second stage, the study-specific estimates were combined using the random-effects model^26,28^. The method of Greenland & Longnecker^27^ was also used for this analysis. A Wald test was used to test whether the non-linear model could be reduced to a linear model.

We used Cochran’s Q test and calculated the *I*^2^ statistic to explore statistical heterogeneity between studies^29^. Furthermore, in order to identify potential sources of heterogeneity, we performed pre-specified subgroup meta-analysis by study characteristics (gender and continent (Asia, Europe and North America)). The random-effects model was used within each subgroup, whereas a fixed-effects model was used across subgroups. The qualitative data synthesis revealed that the age of the study population and the length of follow-up time were rather homogenous across studies, thus, pre-specified subgroup analyses by age at entry (< 50 years, ≥ 50 years) and follow-up time (< 10 years, ≥ 10 years) were not undertaken.

We performed sensitivity analyses excluding studies where > 2 of the quality items in the risk of bias assessment established a “high risk” or “unclear risk” of bias. Furthermore, comparative fixed-effects models were performed as a basis for providing evidence for the necessity of random-effects modelling. Potential publication bias (risk of bias across studies) was assessed both formally with Egger’s test, if at least three studies were available^30^, and visually using funnel plots of the study-specific point risk estimates by the inverse of their standard errors.

Statistical analyses were performed using R, version 3.6.1^31^, with packages dmetar^32^, meta^33^ and dosresmeta^34^. Two-sided *P*-values < 0.05 were considered statistically significant. *P*-values < 0.1 were given with one significant digit.

We used the NutriGrade^35^ scoring system to evaluate the quality of evidence of the linear dose–response meta-analyses (i.e. meta-evidence) for each dairy product subgroup. The NutriGrade scoring system (maximum of 10 points), which has shown good agreement and reliability, includes the following eight items: risk of bias assessment of cohort studies (maximum of 2 points), precision (maximum of 1 point), heterogeneity (maximum of 1 point), directness (maximum of 1 point), publication bias (maximum of 1 point), funding bias (maximum of 1 point), effect size (maximum of 2 points) and dose–response (maximum of 1 point). On the basis of this scoring system, four categories to judge the meta-evidence are recommended: high meta-evidence (≥ 8 points), moderate meta-evidence (6–7.99 points), low meta-evidence (4–5.99 points) and very low meta-evidence (0–3.99 points).

Associations for total intake of dairy products are not readily comparable between studies because total intake of dairy products represents variable products with variable serving sizes. Therefore, data synthesis was constrained to qualitative data synthesis, without a summary RR estimate.

## Results

Out of the 7,768 records identified through database searching and other sources, 88 full-text articles were assessed for eligibility and 33 studies met our eligibility criteria^12–23,36–56^ (see Supplementary Fig. S1 online). The entire list of the 55 full-text articles excluded, with reasons can be found as Supplementary Table S3 online. Among the studies that met our eligibility criteria, three studies^46,49,54^ were subsequently excluded due to duplicate publications (Nurses’ Health Study^12,54^, Caerphilly^13,49^, Health Professionals Follow-up Study^46,55^). In three other studies, there was overlap between the study populations (Malmö Diet and Cancer cohort (one of the Swedish contributions to the European Prospective Investigation Into Cancer and Nutrition (EPIC) cohort)^16^, EPIC-NL cohort (the Dutch contribution to the EPIC cohort)^52^, EPIC^21^) and the exposures considered. The three studies were retained but only the results on the common exposures (milk, yogurt, cheese) from the EPIC cohort^21^ were included in the data synthesis. From the Malmö Diet and Cancer cohort^16^, the results on total dairy and butter were included in the data synthesis, and from the EPIC-NL cohort^52^, the results on total dairy, low-fat dairy and high-fat dairy were included in the data synthesis due to the different exposures than in the EPIC cohort^21^. Thus, 30 studies were included in the qualitative data synthesis^12–23,36–45,47,48,50–53,55,56^ (20 studies for CHD^12,13,16–22,37–40,45,48,50–53,56^ and 12 for ischemic stroke^13–15,23,36,40–44,47,55^). Included in the meta-analysis were 18 studies^12–22,37,38,40,42–45^ (13 studies for CHD^12,13,16–22,37,38,40,45^ and 7 for ischemic stroke^13–15,40,42–44^). No studies on peripheral artery disease were identified.

Table 1 shows the characteristics of the 20 studies on CHD^12,13,16–22,37–40,45,48,50–53,56^. Among the 20 studies, 5 presented gender-specific results^17,20,21,50,53^, 3 comprised only men^13,45,56^ and 3 comprised only women^12,18,40^. A single study was from Asia^22^, 13 from Europe^13,16,18–21,37–39,45,50,52,53^ and 5 from North America^12,17,40,48,56^. A single study comprised 21 countries in 5 continents^51^. The age at entry ranged from 38 to 72 years and the length of follow-up time from 5 to 22 years. All studies except for two of them^22,51^ were classified as being at low risk of bias (see Supplementary Table S4 online).

**Table 1:** Characteristics of the included cohort studies on intake of dairy products and CHD.

First author’s last name and publication year	Cohort name	Study origin	Recruitment year/period	Gender	Age at entry (year)	Sample size	Follow-up (year)	Total number of events	Exposure assessment	Exposure in data synthesis^a^	Ascertainment of outcome	Adjustment variables
Hu 1999	NHS	US	1980	Women	34–59	80,082	14	939	Validated semiquan-titative FFQ	Low-fat dairy products	Nonfatal MI was identified through self-report and confirmed through medical records by study physicians with no knowledge of the participants' self-reported risk factor status. Deaths were identified from state vital records, the National Death Index, next of kin or the postal authorities. Confirmed through hospital records, autopsy report or death certificate. Sudden death within 1 h of the onset of symptoms in women with no other plausible cause of death (other than CHD), were also included	Age, BMI, cigarette smoking, menopausal status, parental history of MI before age 60 y, vitamin E supplement use, alcohol consumption, history of hypertension, aspirin use, vigorous exercise ≥ 1/week, total energy intake, red meat and white meat. Low-fat and high-fat dairy products were entered into the models simultaneously
										High-fat dairy products		
										Low-fat milk		
										High-fat milk		
Al-Delaimy 2003	HPFS	US	1986	Men	40–75	39,800	12	1,458	Validated semiquan-titative FFQ	Total dairy products	Nonfatal MI was identified through self-report and confirmed through medical records. Deaths were identified from the National Death Index or were reported by next of kin, coworkers or the postal authorities. Confirmed through medical records, autopsy report or death certificate	Age, time period, energy intake, history of diabetes, history of hypercholesterolemia, family history of MI, smoking history, aspirin intake, BMI, alcohol intake, physical activity, vitamin E intake, trans fatty acids, ratio of polyunsaturated to saturated fatty acids, total protein intake, cereal fiber, folate, n 3 fatty acids and α-linolenic acid
Elwood 2004	Caerphilly	United Kingdom	1979–83	Men	45–59	2,403	20–24	493	Validated semiquan-titative FFQ	Milk	Fatal CHD and nonfatal MI were identified through self-report and ECG and confirmed through general practitioner and hospital records	Age, total energy intake, smoking, social class, BMI, systolic blood pressure, consumption of alcohol, consumption of fat and prior vascular disease
Buckland 2009	EPIC (the 5 Spanish centers)	Spain	1992–96	Men	29–69	15,335	10.4	480	Validated dietary history question-naire	Total dairy products	CHD events were identified through record linkage of the study population with three sources of information (hospital discharge databases, population-based MI registries and Spanish national and regional mortality registry) and validated. The three sources varied by center	Center, age, education, physical activity, BMI, smoking status, diabetes, hypertension, hyperlipidemia status and total calorie intake
				Women		25,422		126				
Martínez-González 2011	SUN	Spain	1999–2005	Combined	38^b^	13,609	4.9	68	Validated semiquan-titative FFQ	Total dairy products	Nonfatal ACS was identified through self-report and fatal ACS was identified through next of kin, work associates and postal authorities. For participants lost to follow-up, the National Death Index was used to identify deceased cohort members. Outcomes were confirmed through medical records by an expert panel of physicians, blinded to the information on diet and risk factors	Age, gender, family history of CHD, total energy intake, physical activity, smoking, BMI, diabetes at baseline, use of aspirin, history of hypertension and history of hypercholesterolemia
Sonestedt 2011	Malmö Diet and Cancer	Sweden	1991–96	Combined	44–74	26,445	12	1,344	Validated dietary method (7-day menu book, semiquan-titative FFQ and interview)	Total dairy products	CHD events were identified through record linkage of the study population with the Swedish Hospital Discharge Register and the Cause-of-death Register	Age, gender, season, method, energy intake, BMI, smoking, alcohol consumption, leisure-time physical activity and education
										Butter		
Dilis 2012	EPIC-Greece	Greece	1994–99	Men	20–86	9,740	10	426	Validated semiquan-titative FFQ	Total dairy products	CHD events were identified through self-report and confirmed through hospital discharge data, medical records or death certificates	Age, energy intake, BMI, height, physical activity, years of schooling, alcohol consumption, smoking status and arterial blood pressure
				Women		14,189		210				
Avalos 2013	Rancho Bernardo	US	1984–87	Men	50–93	751	20	222	Validated semiquan-titative FFQ	Low-fat milk	Nonfatal MI was identified through self-report. Deaths were identified from death certificates	Age, BMI, diabetes, hypertension and LDL cholesterol
										High-fat milk		
										Yogurt		
										Cheese		
										Low-fat cheese		
										Butter		
				Women		1,008		229		Low-fat milk		Plus current oestrogen use
										High-fat milk		
										Yogurt		
										Cheese		
										Low-fat cheese		
										Butter		
Dalmeijer 2013	EPIC-NL	Nether-lands	1993–97	Combined	49.0 ± 11.9^b^	33,625	13	1,648	Validated semiquan-titative FFQ	Total dairy products	Nonfatal CHD was identified through record linkage of the study population with the Dutch Centre for Health Care Information. Deaths were identified through record linkage with the municipal registries. Causes of death were collected from Statistics Netherlands	Cohort, gender, age, total energy intake, physical activity, smoking, education, BMI, intake of ethanol, coffee, fruit, vegetables, fish, meat and bread
										Low-fat dairy products		
										High-fat dairy products		
Patterson 2013	Swedish Mammo-graphy Cohort	Sweden	1997	Women	48–83	33,636	11.6	1,392	Validated semiquan-titative FFQ	Total dairy products	MI events were identified by linkage of the study population with the Cause of Death Registry and the National Hospital Discharge Registry	Age, smoking status, physical activity, waist-to-hip ratio, alcohol consumption, diagnosis of hypertension, diagnosis of high cholesterol, family history of MI, education, aspirin usage, hormone therapy usage, energy intake and consumption of fruit and vegetables and whole grain foods
										Milk		Plus cultured milk and yogurt, cheese, cream and full-fat crème fraîche and butter
										Low-fat milk		Plus full-fat milk
										High-fat milk		Plus low-fat milk
										Yogurt		Plus milk, cheese, cream and full-fat crème fraîche and butter
										Low-fat yogurt		Plus full-fat cultured milk and yogurt
										High-fat yogurt		Plus low-fat cultured milk and yogurt
										Cheese		Plus milk, cultured milk and yogurt, cream and full-fat crème fraîche and butter
										Low-fat cheese		Plus full-fat cheese
										High-fat cheese		Plus low-fat cheese
Soedamah-Muthu 2013	Whitehall II	United Kingdom	1997–99	Combined	56.1 ± 6.1^b^	4,255	10.8	323	Validated semiquan-titative FFQ	Total dairy products	Nonfatal MI was identified from ECG, questionnaires and doctor's diagnosis of angina and MI and confirmed through medical records. Fatal CHD WAS identified through record linkage of the study population with the National Health Service Central Registry and confirmed through death certificates	Age, ethnicity, employment grade, smoking, alcohol intake, BMI, physical activity, family history of CHD or hypertension, fruit and vegetables, bread, meat, fish, coffee, tea and total energy intake
										Low-fat dairy products		
										High-fat dairy products		
										Milk		
										Yogurt		
										Cheese		
Haring 2014	ARIC	US	1987–89	Combined	45–64	12,066	22	1,147	Validated semiquan-titative FFQ	Total dairy products	Fatal CHD and nonfatal MI were identified using information from study visits, telephone follow-up calls, hospital discharge lists and medical charts, death certificates, next-of-kin and physician-completed questionnaires	Age, gender, race, study, center, total energy intake, smoking, education, systolic blood pressure, use of antihypertensive medication, HDL cholesterol, total cholesterol, use of lipid lowering medication, BMI, waist-to-hip ratio, alcohol intake, sports-related physical activity, leisure-related physical activity, carbohydrate intake, fiber intake and magnesium intake
										Low-fat dairy products		
										High-fat dairy products		
										Low-fat dairy products *for* high-fat dairy products		
Bergholdt 2015	CGPS	Denmark	2003 (ongoing)	Combined	20–100	70,709	5.4	2,777	Semiquan-titative FFQ	Milk	CHD events were identified through record linkage of the study population with the national Danish Patient Registry and the Danish Causes of Death Registry	Gender, age, physical activity in leisure time and at work, smoking, alcohol intake and use of lipid-lowering therapy
Praagman 2015	Rotterdam Study	Nether-lands	1990–93	Combined	> 55	4,235	13.3	567	Validated semiquan-titative FFQ	Total dairy products	Nonfatal MI was identified through digital record linkage with general practitioners and medical specialists in the research area. Trained research assistants checked medical records, including ECG and hospitalization discharge letters. Information on vital status was obtained from municipality records. Causes of death were independently determined by a research physician using information from medical records and subsequently validated by a medical specialist	Age, gender, total energy intake, BMI, smoking, education level and intakes of alcohol, vegetables, fruit, meat, bread, fish, coffee and tea
										Low-fat dairy products		
										High-fat dairy products		
										Yogurt		
										Cheese		
Liu 2017	WHI	US	1994–98	Women	50–79	71,410	13.2	4,229	Validated semiquan-titative FFQ	Butter	CHD events were identified through self-report and confirmed through medical records by a physician	Age, region, race/ethnicity, income, physical activity, BMI, smoking, total energy, hypertension, family history of MI, postmenopausal hormone use, aspirin use and hysterectomy
Dehghan 2018	PURE	21 countries in 5 continents^c^	2003–18	Combined	35–70	136,384	9.1	2,594	Validated FFQ	Total dairy products	MI events were identified through standard case-report forms. Adjudicated centrally in each country by trained physicians using common definitions and supporting documents	Center, age, gender, education, urban or rural location, smoking status, physical activity, history of diabetes, family history of CVD, family history of cancer, fruit, vegetable, red meat, starchy foods and energy
Koskinen 2018	KIHD	Finland	1984–89	Men	42–60	1,981	20.1	472	4-day dietary record (estimation of portion sizes)	Total dairy products	CHD events were identified through record linkage of the study population with the national hospital discharge and death certificate registers	Age, examination year, energy intake, pack-years of smoking, leisure-time physical activity, years of education, family history of CHD, intakes of alcohol, fruits, berries and vegetables, fiber and percentage of energy from polyunsaturated fatty acids
										Cheese		
										Low-fat milk		
										High-fat milk		
										Butter		
Johansson 2019	NSHDS	Sweden	1986–2016	Men	25–75	48,341	14.2	3,102	Validated semiquan-titative FFQ	Milk	MI events were identified through record linkage of the study population with patient and cause of death registers at the National Board of Health and Welfare in Sweden	Gender, age, screening year, BMI, education, physical activity in leisure time, smoking, self-reported family history of CVD or type 2 diabetes, screening project, red meat, whole grain, fruit and vegetables and energy
										Yogurt		
										Cheese		
										Butter		
				Women		50,231		1,193		Milk		
										Yogurt		
										Cheese		
										Butter		
				Combined^d^		98,572		2,009		Low-fat milk		
								1,944		High-fat milk		
								3,037		Low-fat yogurt		
								3,862		High-fat yogurt		
								3,238		Low-fat cheese		
								3,673		High-fat cheese		
Key 2019	EPIC	9 European countries^e^	1992–2000	Combined	Men 52.7 ± 10.3 and women 51.3 ± 9.8^b^	409,885	12.6	7,198	Validated semiquan-titative FFQ in most centers	Milk	Nonfatal MI was identified through a combination of record linkage of the study population with morbidity or hospital registries and self-reports followed by confirmation with medical records. Information on vital status was obtained from mortality registries in most centers except in Greece, where vital status was ascertained through active follow-up of study participants and next of kin. A range of methods was used to confirm the diagnosis of CHD and included medical records, hospital discharge notes, contact with medical professionals, death certificates or verbal autopsy with the next of kin	EPIC center, gender, age, smoking status and number of cigarettes/day, history of diabetes, previous hypertension, prior hyperlipidemia, Cambridge physical activity index, employment status, level of education completed, BMI, current alcohol consumption, observed intakes of energy, fruit and vegetables combined, sugars and fiber from cereals
										Yogurt		
										Cheese		
				Men		106,751		4,608		Milk		Plus red and processed meat, poultry meat, white fish, fatty fish, yogurt, cheese and eggs
										Yogurt		Plus red and processed meat, poultry meat, white fish, fatty fish, milk, cheese and eggs
										Cheese		Plus red and processed meat, poultry meat, white fish, fatty fish, milk, yogurt and eggs
				Women		303,134		2,590		Milk		Plus red and processed meat, poultry meat, white fish, fatty fish, yogurt, cheese and eggs
										Yogurt		Plus red and processed meat, poultry meat, white fish, fatty fish, milk, cheese and eggs
										Cheese		Plus red and processed meat, poultry meat, white fish, fatty fish, milk, yogurt and eggs
Talaei 2019	Isfahan Cohort Study	Iran	2001	Combined	≥ 35	5,432	10.9	564	Validated FFQ	High-fat milk	CHD events were identified through telephone call interview using standard questionnaires. Confirmed through medical records, death certificates, registries, secondary interviews and verbal autopsies	Age, gender, educational level, BMI, physical activity, smoking status, dietary intakes of red meat, poultry, fish, vegetables, fruit, legumes, tea, coffee and non-diet cola and baseline diabetes and hypertension

*ACS* acute coronary syndrome; *ARIC* Atherosclerosis Risk in Communities; *BMI* body mass index; *CGPS* Copenhagen General Population Study; *CHD* coronary heart disease; *CVD* cardiovascular disease; *ECG* electrocardiography; *EPIC* European Prospective Investigation into Cancer and Nutrition; *EPIC-NL* European Prospective Investigation into Cancer and Nutrition-Netherlands; *HDL* high-density lipoprotein; *HPFS* Health Professionals Follow-up Study; *KIHD* Kuopio Ischaemic Heart Disease Risk Factor Study; *LDL* low-density lipoprotein; *MI*,myocardial infarction; *NHS* Nurses' Health Study; *NSHDS* Northern Sweden Health and Disease Study; *PURE* Prospective Urban Rural Epidemiology; *SUN* Seguimiento Universidad de Navarra; *WHI* Women's Health Initiative.

^a^Yogurt defined as yogurt products/other soured milk products.

^b^Mean or mean ± SD.

^c^Argentina, Bangladesh, Brazil, Canada, Chile, China, Colombia, India, Iran, Malaysia, occupied Palestinian territory, Pakistan, Philippines, Poland, South Africa, Saudi Arabia, Sweden, Tanzania, Turkey, United Arab Emirates and Zimbabwe.

^d^Among consumers of low or high fat variants of fermented milk, non-fermented milk or cheese.

^e^Denmark, France, Greece, Italy, Netherlands, Norway, Spain, Sweden and United Kingdom.

Table 2 shows the characteristics of the 12 studies on ischemic stroke^13–15, 23, 36, 40–44, 47, 55^. Among the 12 studies, 1 study presented gender-specific results^55^, 3 comprised only men^13, 15, 23^ and 3 comprised only women^14, 36, 40^. One study was from Asia^41^, five from Europe^13, 15, 42–44^ and six from North America^14, 23, 36, 40, 47, 55^. The age at entry ranged from 46 to 65 years and the length of follow-up time from 8 to 23 years. All studies except for three of them^23, 41, 43^ were classified as being at low risk of bias (see Supplementary Table S4 online).

**Table 2:** Characteristics of the included cohort studies on intake of dairy products and ischemic stroke.

First author’s last name and publication year	Cohort name	Study origin	Recruitment year/period	Gender	Age at entry (year)	Sample size	Follow-up (year)	Total number of events	Exposure assessment	Exposure in data synthesis^a^	Ascertainment of outcome	Adjustment variables
Abbott 1996	Honolulu Heart Program	US	1965–68	Men	55–68	3,150	22	229	Validated 24-h recall	Milk	Identified through hospital discharges, death certificates, autopsy records and at repeated examinations	–
Iso 1999	NHS	US	1980	Women	34–59	85,764	14	347	Validated semiquan-titative FFQ	Milk	Nonfatal stroke was identified through self-report and confirmed by medical records. Fatal stroke was initially ascertained by reports from relatives or postal authorities and a search of the National Death Index and were then confirmed through medical records and death certificates. Medical records were reviewed by physicians blinded to dietary and other risk factors	Age and smoking status
										Yogurt		
										Cheese		
Elwood 2004	Caerphilly	United Kingdom	1979–83	Men	45–59	2,403	20–24	185	Validated semiquan-titative FFQ	Milk	Identified through self-report and ECG and confirmed through general practitioner and hospital records	Age, total energy intake, smoking, social class, BMI, systolic blood pressure, consumption of alcohol, consumption of fat and prior vascular disease
Larsson 2009	ATBC	Finland	1985–88	Men	50–69	26,556 smokers	13.6	2,702	Validated semiquan-titative FFQ	Total dairy products	Identified through record linkage of the study population with the National Hospital Discharge Register and the National Register of Causes of Death. A sample of diagnoses was validated	Age, supplementation group, education, cigarettes smoked daily, BMI, serum total cholesterol, serum HDL cholesterol, histories of diabetes and heart disease, leisure-time physical activity and intakes of total energy, alcohol, caffeine, sugar, red meat, poultry, fish, fruit, fruit juices, vegetables, potatoes, whole grains and refined grains
										Low-fat milk		
										High-fat milk		
										Yogurt		
										Cheese		
										Butter		
Bernstein 2012	HPFS	US	1986	Men	40–75	43,150	22	829	Validated semiquan-titative FFQ	Low-fat dairy products	Nonfatal stroke was identified through self-report and confirmed by medical records. Fatal stroke was identified through state vital records, the National Death Index, next-of-kin or the postal system and confirmed through medical records or autopsy report	Age, time period, BMI, cigarette smoking, physical exercise, parental history of early MI, multivitamin use, vitamin E supplement use, aspirin use at least once per week, total energy, cereal fiber, alcohol, trans-fat, fruit and vegetables and other protein sources
										High-fat dairy products		
	NHS		1980	Women	34–59	84,010	26	1,383		Low-fat dairy products		Plus menopausal status
										High-fat dairy products		
Larsson 2012	Cohort of Swedish Men and Swedish Mammo-graphy Cohort	Sweden	1997	Combined	45–83	74,961	10.2	3,159	Validated semiquan-titative FFQ	Total dairy products	Identified by linkage of the study population with the Swedish Hospital Discharge Registry. Information on dates of deaths was obtained from the Swedish Death Register	Age, gender, smoking status, pack-year of smoking, education, BMI, total physical activity, aspirin use, history of hypertension, diabetes, family history of MI and intakes of total energy, alcohol, coffee, fresh red meat, processed meat, fish, fruits and vegetables
										Low-fat dairy products		Plus full-fat dairy
										High-fat dairy products		Plus low-fat dairy
										Milk		Plus sour milk and yogurt, cheese and cream and crème fraîche
										Yogurt		Plus milk, cheese and cream and crème fraîche
										Cheese		Plus milk, sour milk and yogurt and cream and crème fraîche
Yaemsiri 2012	WHI	US	1994–98	Women	50–79	87,025	7.6	1,049	Validated semiquan-titative FFQ	Total dairy products	Identified through self-report. The potential outcomes were adjudicated locally by physicians and centrally by trained neurologists using additional details from medical charts, brain imaging or death certificates	Age, race, education, family income, years as a regular smoker, hormone replacement therapy use, total MET-hours per week, alcohol intake, history of CHD, history of atrial fibrillation, history of diabetes, aspirin use, use of antihypertensive medication, use of cholesterol-lowering medication, BMI, systolic blood pressure, total energy intake, dietary vitamin E, fruits and vegetable intake and fiber
Lin 2013	CVD-FACTS	Taiwan	1990–93	Combined	45.5 ± 14.2^b^	2,061	12.0	97	Validated semiquan-titative FFQ	Total dairy products	Stroke events before 1996 were identified through self-report and cross-confirmed by medical records or death certificate. Stroke events after 1996 were identified through death certificate data, insurance claim records of the National Health Insurance database and the participant’s self-reported disease history collected in medical records	Gender, baseline age, urinary sodium/creatinine, smoking status, drinking status, physical activity, BMI, systolic blood pressure change, diastolic blood pressure change and hypertension medication
Haring 2015	ARIC	US	1987–89	Combined	45–64	11,601	22.7	598	Validated semiquan-titative FFQ	Total dairy products	Identified through hospital discharge codes and stroke deaths. Confirmed through medical records by physicians	Age, gender, race, study center, total energy intake, smoking, cigarette years, education, systolic blood pressure, use of antihypertensive medication, HDL cholesterol, total cholesterol, use of lipid lowering medication, BMI, waist-to-hip ratio, alcohol intake, sports-related physical activity, leisure-related physical activity, carbohydrate intake, fiber intake, fat intake and magnesium intake
										Low-fat dairy products		
										High-fat dairy products		
Liu 2017	WHI	US	1994–98	Women	50–79	71,410	13.2	1,550	Validated semiquan-titave FFQ	Butter	Identified through self-report and confirmed through medical records by a physician	Age, region, race/ethnicity, income, physical activity, BMI, smoking, total energy, hypertension, family history of MI, postmenopausal hormone use, aspirin use and hysterectomy
Laursen 2018	Diet, Cancer and Health	Denmark	1993–97	Combined	50–64	55,211 without a cancer diagnosis	13.4	1,870	Validated semiquan-titative FFQ	Low-fat milk *for* high-fat milk	Identified through record linkage of the study population with the Danish National Patient Register. The identified cases were verified by review of each individual medical record and/or hospital discharge letter	Total energy intake, age at inclusion, education, BMI, waist circumference adjusted for BMI, smoking, alcohol intake, physical activity and intakes of fruit, vegetables, red meat, processed meat and fish
										Low-fat yogurt *for* high-fat yogurt		
										Low-fat yogurt *for* low-fat milk		
										Low-fat yogurt *for* high-fat milk		
										High-fat yogurt *for* low-fat milk		
										High-fat yogurt *for* high-fat milk		
										Cheese *for* low-fat milk		
										Cheese *for* high-fat milk		
										Cheese *for* low-fat yogurt		
										Cheese *for* high-fat yogurt		
										Cheese *for* butter		
Laursen 2019	EPIC-NL	Netherlands	1993–97	Combined	51.4 (31.1, 63.4)^c^	36,886	15.2	503	Validated semiquan-titative FFQ	Low-fat milk *for* high-fat milk	Identified through record linkage of the study population with a standardised register for hospital discharge diagnoses administered by the Dutch Centre for Health Care Information. Information about vital status was obtained through municipal registries. Causes of death were obtained through linkage with data from Statistics Netherlands	Energy intake, cohort, education, BMI adjusted for waist circumference, smoking, physical activity, alcohol and Dutch Health Diet Index
										Low-fat milk *for* butter		
										High-fat milk *for* butter		
										Low-fat yogurt *for* high-fat yogurt		
										Low-fat yogurt *for* low-fat milk		
										Low-fat yogurt *for* high-fat milk		
										Low-fat yogurt *for* butter		
										High-fat yogurt *for* low-fat milk		
										High-fat yogurt *for* high-fat milk		
										High-fat yogurt *for* butter		
										Cheese *for* low-fat milk		
										Cheese *for* high-fat milk		
										Cheese *for* low-fat yogurt		
										Cheese *for* high-fat yogurt		
										Cheese *for* butter		

*ARIC* Atherosclerosis Risk in Communities; *ATBC* Alpha-Tocopherol, Beta-Carotene Cancer Prevention; *BMI* body mass index; *CHD* coronary heart disease; *CVDFACTS* CardioVascular Disease risk FACtor Two-township Study; *ECG* electrocardiography; *EPIC-NL* European Prospective Investigation into Cancer and Nutrition-Netherlands; *HDL* high-density lipoprotein; *HPFS* Health Professionals Follow-up Study; *MI* myocardial infarction; *MET-hours* metabolic equivalent task-hours; *NHS* Nurses' Health Study; *WHI* Women's Health Initiative.

^a^Yogurt defined as yogurt products/other soured milk products.

^b^Mean ± SD.

^c^Median and 80% central range.

Supplementary Table S5 online shows the definition of dairy products as described in the studies, and Supplementary Table S6 online comprises an overview of which of the studies contributed to each of the meta-analyses. Supplementary Table S7 online shows the funding sources of the studies.

### Total dairy

Overall, the studies indicated no association between total intake of dairy, intake of low-fat dairy or intake of high-fat dairy and risk of CHD or ischemic stroke (see Supplementary Fig. S2-S4 online).

### Milk

**Figure 1:**
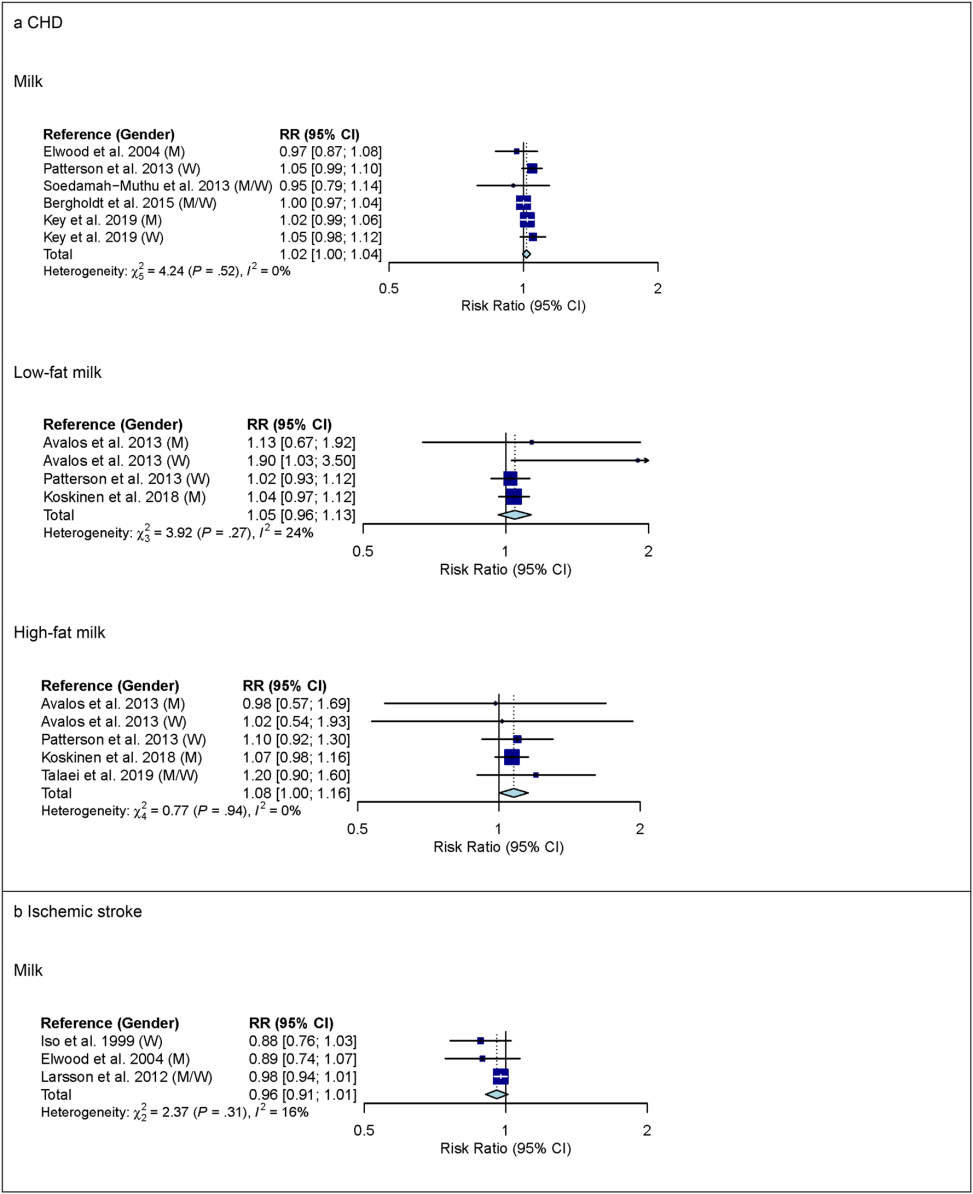
Linear dose–response meta-analysis. Summary RR of CHD (panel **a**) and ischemic stroke (panel **b**) per 200 g higher intake of milk/day. *P* = 0.12 for the association between milk and CHD. *P* = 0.04 for the association between high-fat milk and CHD. *CHD* coronary heart disease; *CI* confidence interval; *M* males; *RR* risk ratio; *W* women.

For milk, 6 studies with 619,460 participants and 16,478 cases were included in the high versus low intake meta-analysis for CHD (overall intake range: 0–710 g/d). Comparing the highest with the lowest category of milk intake, no association was observed for CHD (RR 1.02 (95% CI 0.92–1.13); *I*^2^ = 67%; *P*_*heterogenity*_ (*P*_*het*_) < 0.01) (see Supplementary Fig. S5 online). Comparing the highest with the lowest category of low-fat milk intake, no association with CHD was observed (RR 1.05 (95% CI 0.92–1.20); *I*^2^ = 59%; *P*_*het*_ = 0.03; n studies = 5), whereas comparing the highest with the lowest category of high-fat milk intake, a higher risk of CHD was observed (RR 1.16 (95% CI 1.01–1.33); *I*^2^ = 53%; *P*_*het*_ = 0.04; n studies = 6) (see Supplementary Fig. S5 online). The observed heterogeneity between studies was not explained by pre-specified subgroup analyses (see Supplementary Table S8 online). In linear dose–response meta-analysis, no associations were observed between milk intake or low-fat milk intake and CHD (Fig. 1). However, each additional daily 200 g of high-fat milk was associated with an 8% higher risk of CHD (RR 1.08 (95% CI 1.00–1.16); *I*^2^ = 0%; *P*_*het*_ = 0.94; n studies = 4) (Fig. 1). Supplementary Table S9 online shows subgroup dose–response meta-analysis by study characteristics. No evidence of non-linear dose–response associations was observed (data not shown).

Three studies with 163,128 participants and 3,691 cases were included in the high versus low intake meta-analysis for milk and ischemic stroke (overall intake range: 0–710 g/day). Comparing the highest with the lowest category of milk intake, a lower risk of ischemic stroke was observed (RR 0.88 (95% CI 0.79–0.98); *I*^2^ = 0%; *P*_*het*_ = 0.52) (see Supplementary Fig. S5 online). In linear dose–response meta-analysis, no association between milk intake and ischemic stroke was observed (Fig. 1). Supplementary Tables S8 and S9 online show subgroup meta-analysis by study characteristics. No evidence of a non-linear dose–response association was observed (data not shown).

### Yogurt

**Figure 2:**
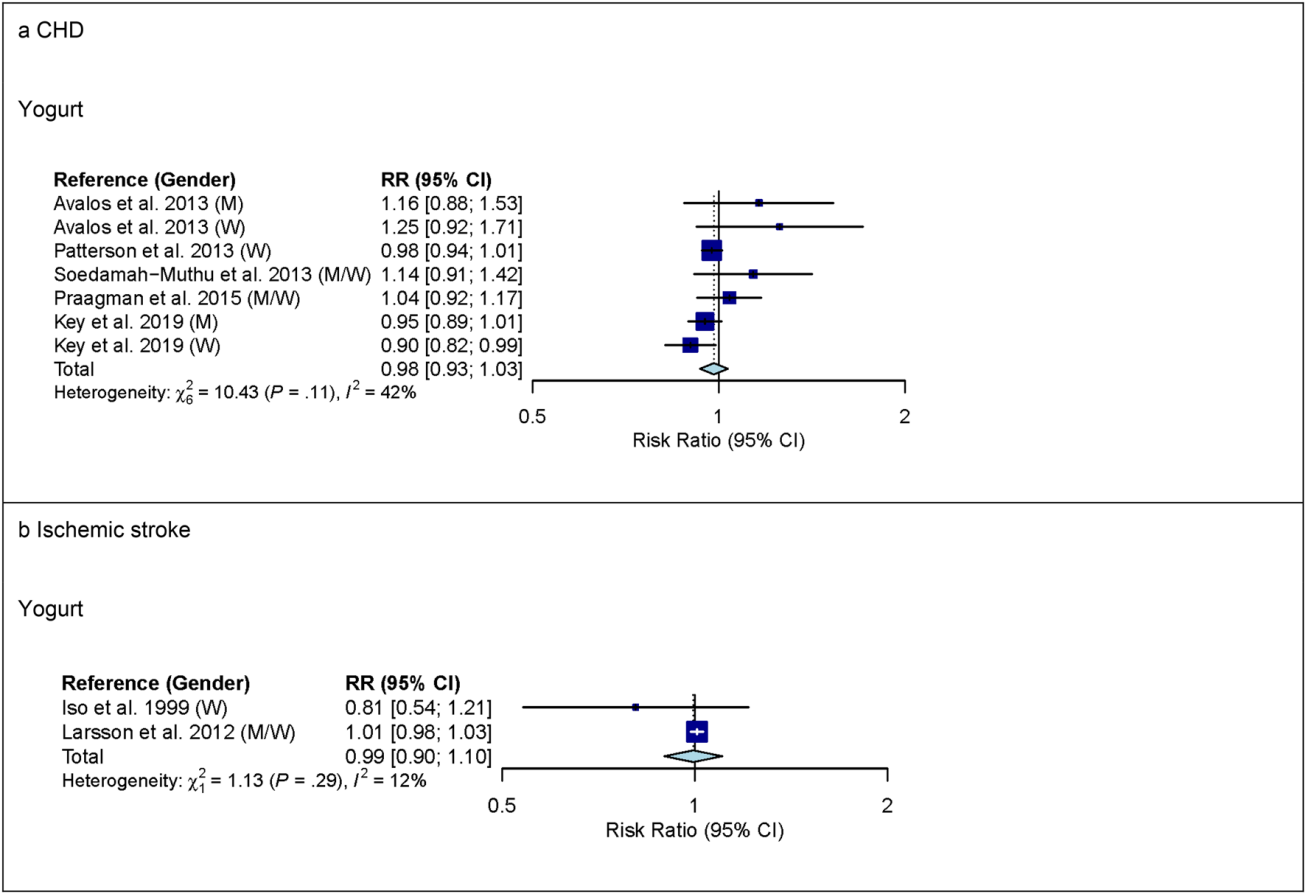
Linear dose–response meta-analysis. Summary RR of CHD (panel **a**) and ischemic stroke (panel **b**) per 100 g higher intake of yogurt/day. Yogurt defined as yogurt/other soured milk products. *CHD* coronary heart disease; *CI* confidence interval; *M* males; *RR* risk ratio; *W* women.

For yogurt, 6 studies with 552,342 participants and 14,226 cases were included in the high versus low intake meta-analysis for CHD (overall intake range: 0–440 g/day). Comparing the highest with the lowest category of yogurt intake, no association with CHD was observed (RR 0.99 (95% CI 0.91–1.08); *I*^2^ = 49%; *P*_*het*_ = 0.06) (see Supplementary Fig. S6 online). In pre-specified subgroup analysis by continent, however, heterogeneity between continents was observed (*P* < 0.05) but no association between yogurt and CHD was observed in studies from Europe (RR 0.96 (95% CI 0.89–1.04); *I*^2^ = 42%; *P*_*het*_ = 0.12; n studies = 5) or in studies from North America (RR 1.25 (95% CI 0.97–1.61); *I*^2^ = 0%; *P*_*het*_ = 0.71; n studies = 1) (see Supplementary Table S10 online). No association between low-fat or high-fat yogurt and CHD was observed (see Supplementary Fig. S6 online). In linear dose–response meta-analysis, no association between yogurt and CHD was observed (RR 0.98 (95% CI 0.93–1.03) per 100 g higher intake per day; *I*^2^ = 42%; *P*_*het*_ = 0.11; n studies = 5) (Fig. 2). However, heterogeneity between continents was observed (*P* < 0.05) but no association between yogurt and CHD was observed in studies from Europe (RR 0.97 (95% CI 0.93–1.01) per 100 g higher intake per day; *I*^2^ = 35%; *P*_*het*_ = 0.19; n studies = 4) or in studies from North America (1.20 (95% CI 0.98–1.48) per 100 g higher intake per day; *I*^2^ = 0%; *P*_*het*_ = 0.71; n studies = 1) (see Supplementary Table S11 online). No evidence of a non-linear dose–response association was observed (data not shown).

Three studies with 187,281 participants and 6,208 cases were included in the high versus low intake meta-analysis for yogurt and ischemic stroke (overall intake range: 0–400 g/day). Comparing the highest with the lowest category of yogurt intake, no association for ischemic stroke was observed (see Supplementary Fig. S6 online). Supplementary Table S10 online shows subgroup high versus low intake meta-analysis by study characteristics. Also in linear dose–response meta-analysis, no association between yogurt intake and ischemic stroke was observed (Fig. 2).

### Cheese

**Figure 3:**
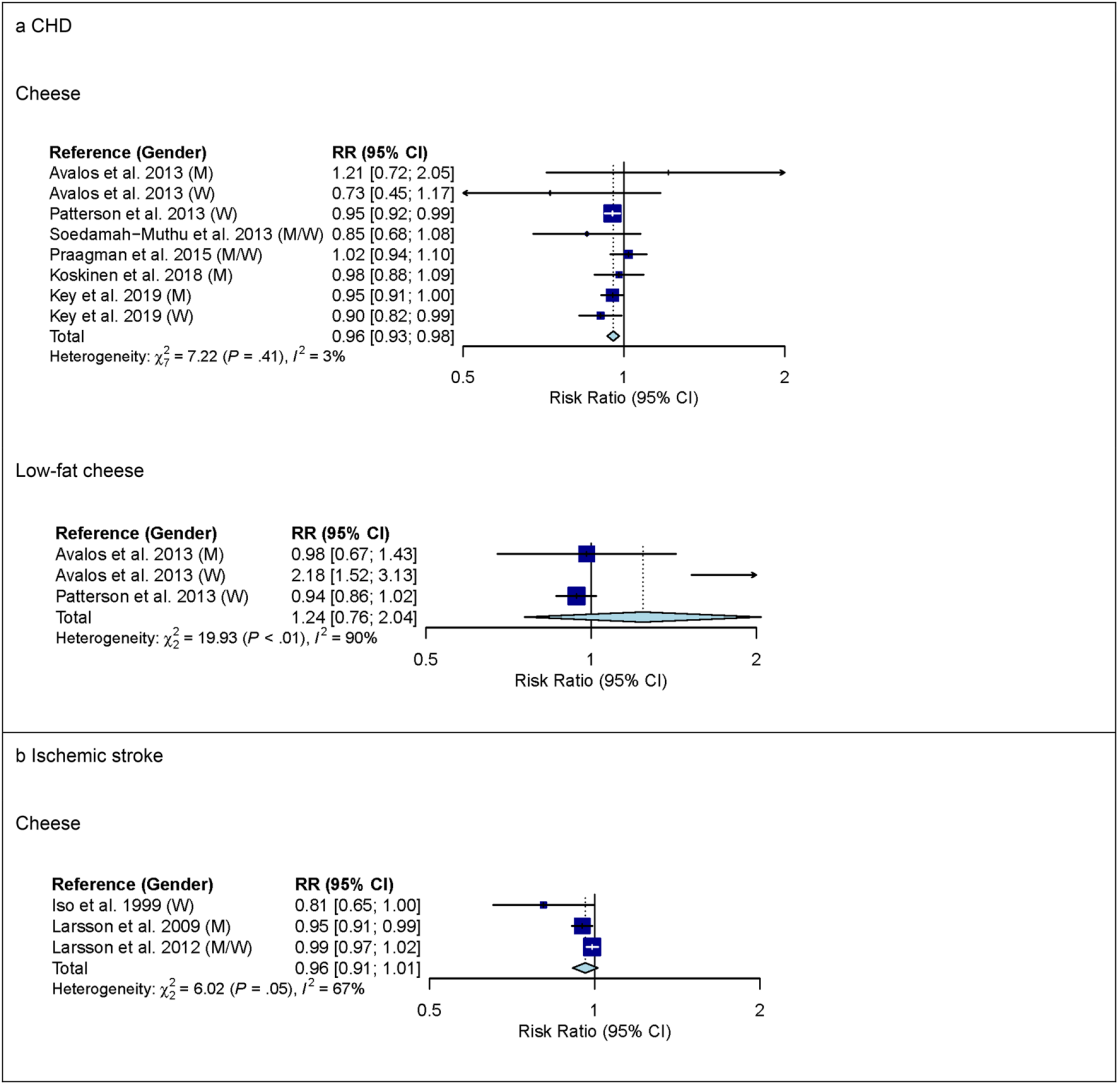
Linear dose–response meta-analysis. Summary RR of CHD (panel **a**) and ischemic stroke (panel **b**) per 20 g higher intake of cheese/day. *P* < 0.05 for heterogeneity for the association between cheese and ischemic stroke. *CHD* coronary heart disease; *CI* confidence interval; *M* males; *RR* risk ratio; *W* women.

For cheese, 7 studies with 554,323 participants and 14,698 cases were included in the high versus low intake meta-analysis for CHD (overall intake range: 0–120 g/day). Comparing the highest with the lowest category of cheese intake, a lower risk of CHD was observed (RR 0.91 (95% CI 0.84–0.99); *I*^2^ = 37%; *P*_*het*_ = 0.12) (see Supplementary Fig. S7 online). In pre-specified subgroup analysis by gender, however, heterogeneity between genders was observed (*P* = 0.03) (see Supplementary Table S12 online). In studies among men, comparing the highest with the lowest category of cheese intake, no association with CHD was observed (RR 1.03 (95% CI 0.93–1.13); *I*^2^ = 0%; *P*_*het*_ = 0.74; n studies = 3). In studies among women, comparing the highest with the lowest category of cheese intake, a lower risk of CHD was observed (RR 0.82 (95% CI 0.69–0.97); *I*^2^ = 33%; *P*_*het*_ = 0.23; n studies = 3). No association between low-fat or high-fat cheese intake and CHD was observed but heterogeneity for low-fat cheese intake was observed (see Supplementary Fig. S7 online). The observed heterogeneity between studies was not explained by subgroup analyses (see Supplementary Table S12 online). In linear dose–response meta-analysis, each additional daily 20 g of cheese was associated with a 4% lower risk of CHD (RR 0.96 (95% CI 0.93–0.98); *I*^2^ = 3%; *P*_*het*_ = 0.41; n studies = 6) (Fig. 3) and no heterogeneity between genders was observed (*P* = 0.55) (see Supplementary Table S13 online). In studies among men, the summary RR estimate for cheese intake of 20 g/day was 0.96 (95% CI 0.92–1.00) and in studies among women, the summary RR estimate for cheese intake of 20 g/day was 0.94 (95% CI 0.90–0.98). No association between low-fat cheese intake and CHD was observed but heterogeneity was observed (Fig. 3). The observed heterogeneity between studies was not explained by subgroup analyses (see Supplementary Table S13 online). No evidence of a non-linear dose–response association was observed (data not shown).

Three studies with 187,281 participants and 6,208 cases were included in the high versus low intake meta-analysis for cheese and ischemic stroke (overall intake range: 0–400 g/day). Comparing the highest with the lowest category of cheese intake, no association with ischemic stroke was observed (see Supplementary Fig. S7 online). Supplementary Table S12 online shows subgroup high versus low intake meta-analysis by study characteristics. Also in linear dose–response meta-analysis, no association between cheese intake and ischemic stroke was observed but heterogeneity was observed (Fig. 3). The observed heterogeneity between studies was not explained by subgroup analyses (see Supplementary Table S13 online). No evidence of a non-linear dose–response association was observed (data not shown).

### Butter

**Figure 4:**
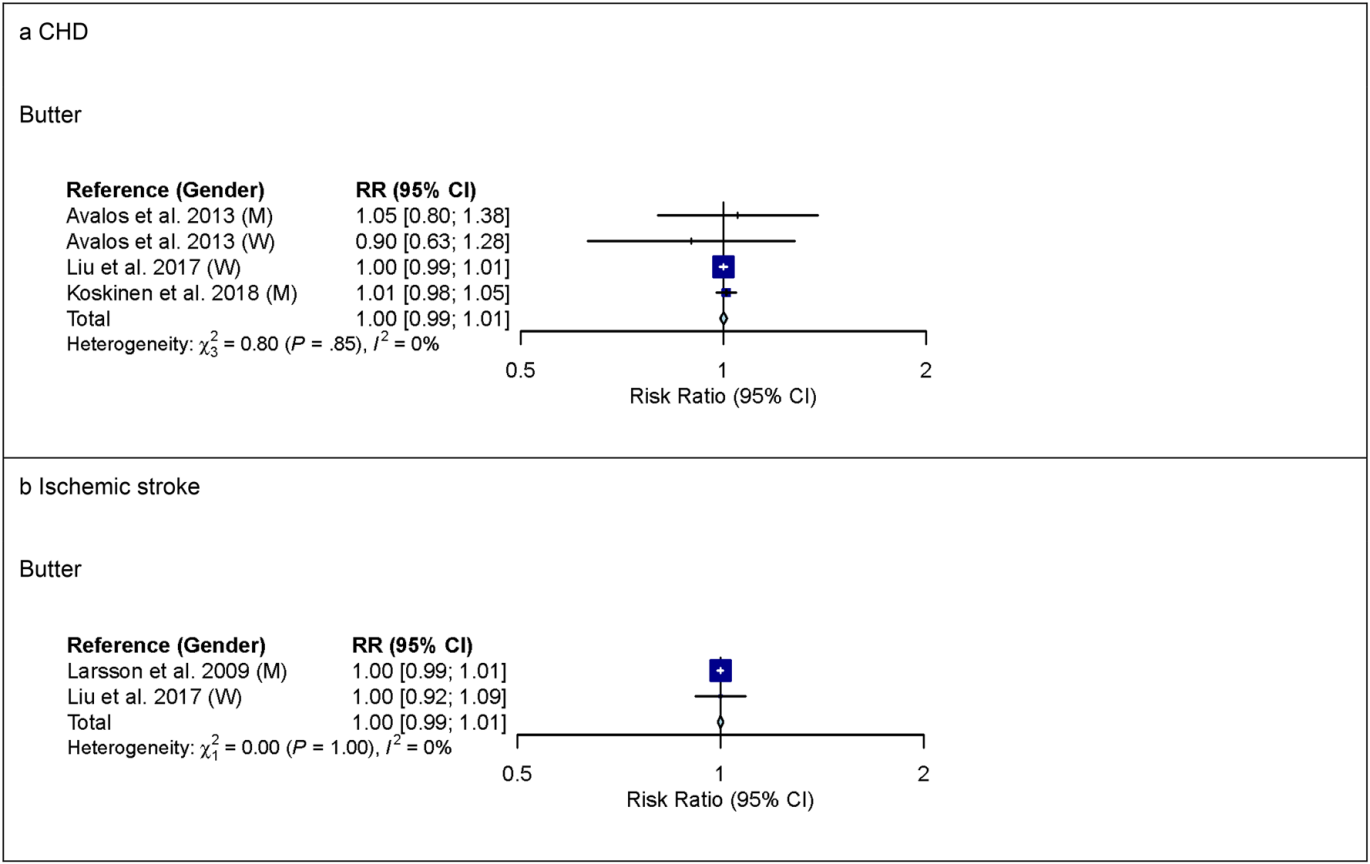
Linear dose–response meta-analysis. Summary RR of CHD (panel **a**) and ischemic stroke (panel **b**) per 6 g higher intake of butter/day. *CHD* coronary heart disease; *CI* confidence interval; *M* males; *RR* risk ratio; *W* women.

For butter, 4 studies with 128,757 participants and 6,562 cases were included in the high versus low intake meta-analysis for CHD (overall intake range: 0–63 g/day). Comparing the highest with the lowest category of butter intake, no association was observed for CHD (see Supplementary Fig. S8 online). Also in linear dose–response meta-analysis, no association between butter intake and CHD was observed (Fig. 4). Supplementary Tables S14 and S15 online show subgroup meta-analysis by study characteristics.

In linear dose–response meta-analysis, no association between butter intake and ischemic stroke was observed (Fig. 4).

### Substitutions between dairy product subgroups

**Figure 5:**
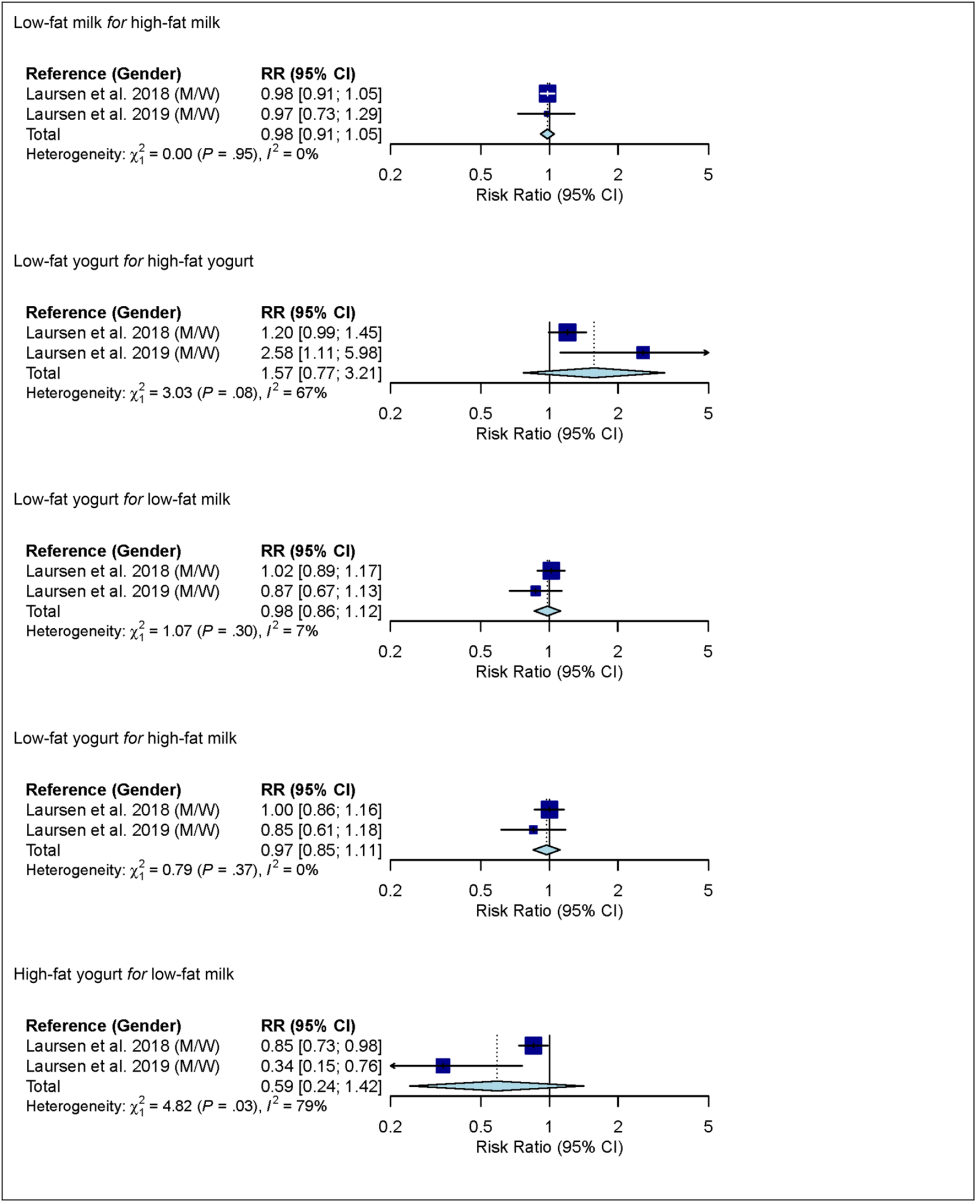

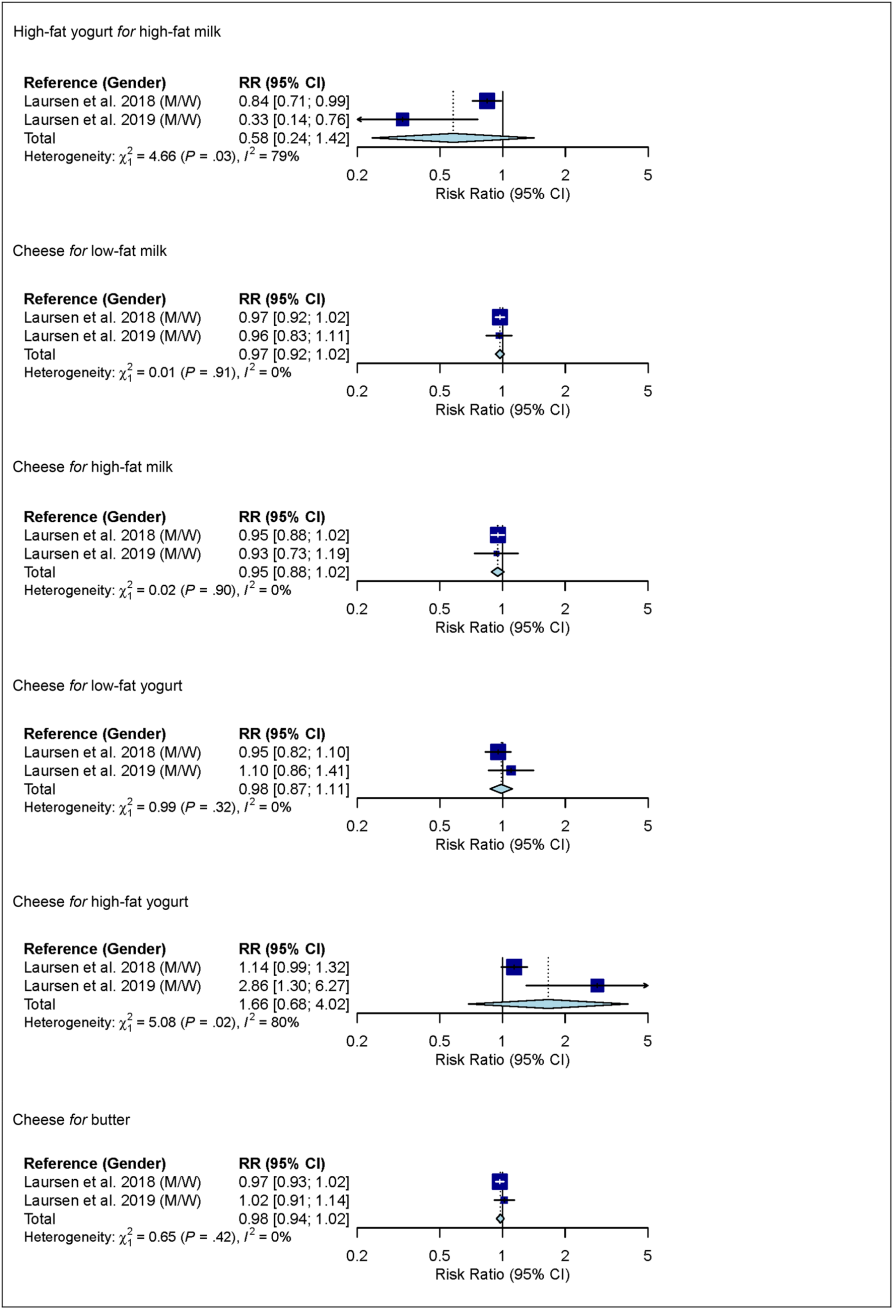
Linear dose–response meta-analysis. Summary RR of ischemic stroke for substitutions between dairy product subgroups (per 1 serving/day). For example in the mean of substitution of low-fat milk *for* high-fat milk; i.e. low-fat milk takes the place of high-fat milk). For milk and yogurt, the serving size was 200 g, for cheese 20 g, and for butter 6 g. Yogurt defined as yogurt/other soured milk products. *CI* confidence interval; *M* males; *RR* risk ratio; *W* women.

In linear dose–response meta-analysis, no associations between substitutions among dairy product subgroups (low-fat milk, high-fat milk, low-fat yogurt, high-fat yogurt, cheese and butter) and risk of ischemic stroke were observed (Fig. 5).

### Sensitivity analyses

One study^22^, investigating high-fat milk intake and risk of CHD, was excluded in low risk of bias sensitivity analysis. The reanalyses produced small changes in the summary RR estimates and corresponding 95% CIs (see Supplementary Fig. S9 online). Using a fixed-effects model to calculate summary RR estimates and corresponding 95% CIs produced narrower CIs, and eight of the summary RR estimates became statistically significant (see Supplementary Table S16 online). Among these analyses, heterogeneity was observed in four analyses; thus, the narrower CIs from the fixed-effects model were not considered reliable. In four other analyses, no heterogeneity was observed. In these analyses, except for one, the fixed-effects model produced borderline statistical significance with CIs including 0.99. The exception was the linear dose–response meta-analysis of substitution of low-fat yogurt for high-fat yogurt where the fixed-effects model produced statistical significance with a CI of 1.03–1.50 while Cochran’s Q-test for heterogeneity was borderline statistically significant with *P* = 0.08 (I^2^ = 67%). In that case, we deem the analysis inconclusive. There was no evidence of publication bias with the exception of high versus low intake meta-analysis of yogurt and risk of CHD (Egger’s test: *P* < 0.01). Visual inspection of funnel plots suggested moderate small study effects towards the null for yogurt intake and risk of CHD in both high versus low intake and dose–response meta-analysis (see Supplementary Fig. S10 online).

### Quality of meta-evidence

Table 3 provides an overview of the findings from linear dose–response meta-analysis on dairy product subgroups and risk of CHD, and NutriGrade meta-evidence grading. The grading was rated *moderate* for high-fat milk and cheese, and *low* for milk, low-fat milk, yogurt, low-fat cheese and butter.

**Table 3:** Linear dose–response meta-analysis on dairy product subgroups and risk of coronary heart disease, and NutriGrade meta-evidence grading.

Dairy product^a^	Amount	Number of studies	Summary RR^b^	95% CI	I^2^ (%)	NutriGrade grading grading
Milk	Per 200 g	5	1.02	1.00, 1.04^c^	0	Low^d^
Low-fat milk	Per 200 g	3	1.05	0.96, 1.13	24	Low
High-fat milk	Per 200 g	4	1.08	1.00, 1.16^e^	0	Moderate^f^
Yogurt	Per 100 g	5	0.98	0.93, 1.03	42	Low
Low-fat yogurt	–	–	–	–	–	–
High-fat yogurt	–	–	–	–	–	–
Cheese	Per 20 g	6	0.96	0.93, 0.98	3	Moderate
Low-fat cheese	Per 20 g	2	1.24	0.76, 2.04	90	Low
High-fat cheese	–	–	–	–	–	–
Butter	Per 6 g	3	1.00	0.99, 1.01	0	Low

*CI* confidence interval; *RR* risk ratio.

^a^Yogurt defined as yogurt/other soured milk products.

^b^Scoring for effects size was based on summary RR estimates from dose–response meta-analysis.

^c^*P* = 0.12.

^d^There is low confidence in the effect estimate; further research will provide important evidence on the confidence and likely change the effect estimate.

^e^*P* = 0.04.

^f^There is moderate confidence in the effect estimate, further research could add evidence on the confidence and may change the effect estimate.

Table 4 provides an overview of the findings from linear dose–response meta-analysis on dairy product subgroups and risk of ischemic stroke, and NutriGrade meta-evidence grading. The grading was rated *low* for milk, yogurt, cheese and butter, and *very low* for all substitutions between dairy product subgroups.

**Table 4:** Linear dose–response meta-analysis on dairy product subgroups and risk of ischemic stroke, and NutriGrade meta-evidence grading.

Dairy product^a^	Amount	Number of studies	Summary RR^b^	95% CI	I^2^ (%)	NutriGrade grading
Milk	Per 200 g	3	0.96	0.91, 1.01	16	Low^c^
Low-fat milk	–	–	–	–	–	–
High-fat milk	–	–	–	–	–	–
Yogurt	Per 100 g	2	0.99	0.90, 1.10	12	Low
Low-fat yogurt	–	–	–	–	–	–
High-fat yogurt	–	–	–	–	–	–
Cheese	Per 20 g	3	0.96	0.91, 1.01	67	Low
Low-fat cheese	–	–	–	–	–	–
High-fat cheese	–	–	–	–	–	–
Butter	Per 6 g	2	1.00	0.99, 1.01	0	Low
Low-fat milk *for* high-fat milk^d^	Per 1 serving/day	2	0.98	0.91, 1.05	0	Very low^e^
Low-fat yogurt *for* high-fat yogurt	Per 1 serving/day	2	1.57	0.77, 3.21	67	Very low
Low-fat yogurt *for* low-fat milk	Per 1 serving/day	2	0.98	0.86, 1.12	7	Very low
Low-fat yogurt *for* high-fat milk	Per 1 serving/day	2	0.97	0.85, 1.11	0	Very low
High-fat yogurt *for* low-fat milk	Per 1 serving/day	2	0.59	0.24, 1.42	79	Very low
High-fat yogurt *for* high-fat milk	Per 1 serving/day	2	0.58	0.24, 1.42	79	Very low
Cheese *for* low-fat milk	Per 1 serving/day	2	0.97	0.92, 1.02	0	Very low
Cheese *for* high-fat milk	Per 1 serving/day	2	0.95	0.88, 1.02	0	Very low
Cheese *for* low-fat yogurt	Per 1 serving/day	2	0.98	0.97, 1.11	0	Very low
Cheese *for* high-fat yogurt	Per 1 serving/day	2	1.66	0.68, 4.02	80	Very low
Cheese *for* butter	Per 1 serving/day	2	0.98	0.94, 1.02	0	Very low

*CI* confidence interval; *RR* risk ratio.

^a^Yogurt defined as yogurt/other soured milk products.

^b^Scoring for effects size was based on summary RR estimates from dose–response meta-analysis.

^c^There is low confidence in the effect estimate, further research will provide important evidence on the confidence and likely change the effect estimate.

^d^Substitutions between dairy product subgroups (per 1 serving/day). For example in the mean of substitution of low-fat milk *for* high-fat milk; i.e. low-fat milk takes the place of high-fat milk. For milk and yogurt, the serving size was 200 g, for cheese 20 g, and for butter 6 g.

^e^There is very low confidence in the effect estimate; meta-evidence is very limited and uncertain.

## Discussion

Intake of dairy product subgroups and associations with major atherosclerotic CVDs in the general adult population was investigated in this systematic review and meta-analysis of cohort studies through comparison of the highest with the lowest intake categories and dose–response (linear and non-linear) analyses. Intake of high-fat milk was positively associated with the risk of CHD, with heterogeneity present in high versus low intake meta-analysis, which could not be explained by pre-specified subgroup analyses. However, no heterogeneity was observed in linear dose–response meta-analysis. For total intake of milk and intake of low-fat milk, no associations were observed. Intake of cheese was inversely associated with the risk of CHD, with heterogeneity between genders present in high versus low intake meta-analysis. In studies among women, high cheese intake compared with low cheese intake was associated with lower CHD risk but not in studies among men. However, no heterogeneity between genders was observed in linear dose–response meta-analysis. For intake of low-fat cheese, yogurt and butter, no associations with CHD were observed. Milk intake was inversely associated with the risk of ischemic stroke in high versus low intake meta-analysis but not in dose–response meta-analysis. For intake of cheese, yogurt and butter no associations with ischemic stroke were observed. The NutriGrade tool for evaluating the quality of meta-evidence suggested a *moderate* confidence in the summary RR estimates for the associations between high-fat milk and cheese and CHD; further research could add evidence on the confidence and may change the effect estimates. For all other summary RR estimates, the tool suggested a *low* confidence in the effect estimates (further research will provide important evidence on the confidence and likely change the estimates) or a *very low* confidence in the effect estimates (meta-evidence is very limited and uncertain).

For total intake of dairy, intake of low-fat dairy and intake of high-fat dairy, data synthesis was constrained to qualitative data synthesis without a summary RR estimate. Overall, these studies indicated no association between total intake of dairy, intake of low-fat dairy or intake of high-fat dairy and CHD or ischemic stroke.

The strengths of our systematic review are the direct evidence, the focus on *atherosclerotic* CVDs and the inclusion of only studies considering total CHD and total ischemic stroke as outcomes. Previous meta-analyses on dairy intake and CHD and stroke combined results from studies on total and fatal outcomes^6–9^. Moreover, our data synthesis was also differentiated according to the fat content of the dairy product subgroups. Whole-fat dairy products have a high saturated fat content and nutrient trials have shown that intake of saturated fatty acids increases serum LDL-cholesterol relative to intake of unsaturated fatty acids and carbohydrates^2^. Elevated LDL-cholesterol is an important risk factor of atherosclerosis, which is the primary cause of atherosclerotic CVDs^1^. In addition, we carefully considered dairy product subgroups and contacted authors of the included studies for further information on exposure definition. The quality of a systematic review and meta-analysis depends on the quality of the included studies. The NOS^24^ is a commonly used tool for quality ranking of cohort studies. However, the NOS tool does not focus on internal validity alone as also was emphasized by Stang^57^. We employed a modified instrument based on the NOS tool. We assessed the quality of the included studies by assessing eight items concerning the internal validity, categorized into three domains (selection bias, information bias and comparability) similar to the domains in the NOS tool. Studies were classified as being at low risk of bias in general if only ≤ 2 of the 8 quality items established a “high risk” or “unclear risk” of bias. In sensitivity analyses, we repeated the meta-analyses including only studies deemed low risk of bias. The reanalyses produced small changes in the CIs; thus, the confidence limits appeared to be insensitive to judgements about study quality. We did not include selective reporting of outcome and analyses in our assessment of the quality of the cohort studies because most cohort studies are not registered before being conducted. We performed comparative fixed-effects modelling to evaluate the robustness of the results, which provided evidence for the necessity of random-effects modelling. Finally, we also evaluated the meta-evidence for each dairy subgroup using a comprehensive approach; namely the NutriGrade^35^ scoring system.

Limitations of our systematic review are that the number of included studies was low, limiting the possibility to detect heterogeneity for several exposures and to identify potential sources of heterogeneity in subgroup analyses by study characteristics. The qualitative data synthesis revealed that the age at entry of the study populations (≥ 50 years in all studies, except for 2 studies) and length of follow-up (≥ 10 years, except for 1 study) were homogenous across studies but sources of bias were present and these biases varied across the studies as demonstrated in our risk of bias judgement. For example, socioeconomic status is a risk factor of CVD^58, 59^ and low-fat dairy products are more likely to be consumed by groups of higher socioeconomic status^60^. However, most studies adjusted for education and other relevant risk factors of atherosclerotic CVD such as gender (as appropriate), age, total energy intake, smoking, physical activity, alcohol consumption, body mass index and comorbidity. We observed no evidence of publication bias with the exception of high versus low intake meta-analysis of yogurt and risk of CHD (Egger’s test: *P* < 0.01). However, large *P*-values do not indicate that publication bias can be safely ignored. In both high versus low intake and dose–response meta-analysis, visual inspection of funnel plots suggested moderate small study effects towards the null for yogurt and risk of CHD, due to the right-skewed form of the funnel plots. We considered the two bibliographic databases PubMed and Embase to be the most important information sources to search studies. We did not supplement our search by consulting other experts in the field as unpublished studies may tend to be of lower quality and because only a biased sample of such studies can be identified^25^. On the other hand, it can be argued that inclusion of only published studies may introduce publication bias^25^. Furthermore, the lack of searching non-English databases may be a source of publication bias^61^. We did not search grey literature (such as reports), because grey literature may not have been subject to peer review and therefore may be of lower quality. Searching study registries was not considered relevant. Until recently, specified food substitutions have not been addressed in cohort studies on dairy and CVDs. Thus, summarizing findings on substitutions between dairy products were possible from only two studies. The lack of specifying substitutions in most previous cohort studies has implications for the interpretation of the results of our systematic review and meta-analysis as the effect of a specific dairy product on CVD depends on the replaced foods^62^.

Guo et al.^9^ summarized findings of cohort studies on the associations between total intake of milk (11 studies), total intake of yogurt (3 studies) and cheese (9 studies) and the risk of CHD and observed no associations in their systematic review from 2017. The meta-analysis on milk intake and CHD risk was subsequently updated with one study by Soedamah-Muthu & de Goede^63^. In agreement with previous findings, no association between total intake of milk and CHD was observed. Also, we did not observe any association for total intake of milk or yogurt with CHD. But we observed an inverse association between intake of cheese and risk of CHD in agreement with the findings by Chen et al.^8^ in their meta-analysis of eight prospective observational studies on cheese intake and CHD risk from 2017. The effect of cheese intake on blood lipids was reviewed and summarized in 2015 by de Goede et al.^64^. The authors found that intake of hard cheese decreased total cholesterol, LDL-cholesterol and high-density lipoprotein (HDL)-cholesterol when compared with intake of butter of a similar ratio of polyunsaturated fatty acids to saturated fatty acids, and speculated that calcium, specific types of saturated fatty acids or the food matrix may explain the findings. However, in a cohort study (published after we finished our literature search), no association between substitution of cheese for butter was observed^65^. In line with the meta-analysis of three prospective observational studies on butter intake and CHD risk from 2016 by Pimpin et al.^7^, we observed no association between butter intake and CHD risk. Associations for low- and high-fat milk intake with major atherosclerotic CVDs have not been considered in previous systematic reviews. We observed that higher intake of high-fat milk was associated with higher risk of CHD. For intake of low-fat milk, no association was observed. High-fat milk has a high saturated fat content that may partly explain the findings of a higher risk of CHD associated with a higher intake of high-fat milk. Results from dietary trials have shown that whole-fat milk increases total cholesterol and LDL-cholesterol more than low-fat milk such as skim milk^66^. However, the effects of whole-fat milk on HDL-cholesterol and the ratio of total cholesterol to HDL-cholesterol were less clear^66^.

de Goede et al.^6^ summarized findings of cohort studies on the association between intake of dairy products and risk of ischemic and hemorrhagic stroke aggregated in their systematic review from 2016. In subgroup analyses by stroke type (ischemic and hemorrhagic), associations with total intake of milk were investigated. No association between total intake of milk and risk of ischemic stroke (5 studies) was observed, but the 95% CI was compatible with meaningful benefits in line with our findings. In addition, no association between yogurt, cheese or butter and ischemic stroke was observed in our meta-analysis.

In conclusion, this systematic review and meta-analysis of cohort studies indicates a positive association between high-fat milk and the risk of CHD, with heterogeneity present in high versus low intake meta-analysis but not in linear dose–response meta-analysis. The observed heterogeneity in high versus low intake meta-analysis could not be explained by pre-specified subgroup analyses. In addition, this systematic review and meta-analysis of cohort studies indicates an inverse association between intake of cheese and the risk of CHD, with heterogeneity present in high versus low intake meta-analysis but not in linear dose–response meta-analysis. The observed heterogeneity in high versus low intake meta-analysis could partly be explained by gender. The NutriGrade meta-evidence grading was rated *moderate* for the associations between high-fat milk and cheese and the risk of CHD. For all other associations between dairy product subgroups and risk of CHD or risk of ischemic stroke, the meta-evidence grading was rated *low* or *very low*. No studies on peripheral artery disease were identified. Studies with more details about types of dairy products, including fat content, are warranted. Furthermore, future studies should investigate substitutions between dairy product subgroups. Findings from dairy food substitution analyses are important in deriving food-based dietary guidelines. Finally, future studies should describe their methods and data in as much detail as feasible, to facilitate later systematic reviews and meta-analyses.

## Supplementary Information

Supplementary Information

## Data Availability

The dataset generated and analysed during the current review is available from the corresponding author on reasonable request.

## Code availability

The computer code is available at GitHub (https://github.com/DTUComputeStatisticsAndDataAnalysis/Intake-of-dairy-products-and-associations-with-major-atherosclerotic-cardiovascular-diseases).
