# Supplementary material for: Intake of dairy products and associations with major atherosclerotic cardiovascular diseases: a systematic review and meta-analysis of cohort studies
Source: Sci Rep. 2021 Jan 14;11:1303. doi: 10.1038/s41598-020-79708-x (PMC7809206; doi:10.1038/s41598-020-79708-x)
Supplement: Supplementary file 1 — Supplementary Information [file 41598_2020_79708_MOESM1_ESM.pdf]

Supplementary information

Intake of dairy products and associations with major atherosclerotic cardiovascular diseases: A systematic review and meta-analysis of cohort studies

Marianne Uhre Jakobsen*, Ellen Trolle, Malene Outzen, Heddie Mejborn, Manja G. Grønberg, Christian Bøge Lyndgaard, Anders Stockmarr, Stine K. Venø, Anette Bysted

*Correspondence to muja@food.dtu.dk

Supplementary Table S1	PRISMA checklist
Supplementary Table S2	Conversion of serving size to grams
Supplementary Table S3	Full-text articles excluded, with reasons
Supplementary Table S4	Risk of bias judgements of included studies
Supplementary Table S5	Definition of dairy products as described in included studies
Supplementary Table S6	Overview of which of the included studies contributed to each of the meta-analyses
Supplementary Table S7	Funding sources of included studies
Supplementary Table S8	High versus low intake meta-analysis for milk and coronary heart disease and ischemic stroke by gender and continent
Supplementary Table S9	Linear dose-response meta-analysis for milk intake and coronary heart disease and ischemic stroke by gender and continent
Supplementary Table S10	High versus low intake meta-analysis for yogurt and coronary heart disease and ischemic stroke by gender and continent
Supplementary Table S11	Linear dose-response meta-analysis for yogurt intake and coronary heart disease by gender and continent
Supplementary Table S12	High versus low intake meta-analysis for cheese and coronary heart disease and ischemic stroke by gender and continent
Supplementary Table S13	Linear dose-response meta-analysis for cheese intake and coronary heart disease and ischemic stroke by gender and continent
Supplementary Table S14	High versus low intake meta-analysis for butter and coronary heart disease by gender and continent
Supplementary Table S15	Linear dose-response meta-analysis for butter intake and coronary heart disease by gender and continent
Supplementary Table S16	Meta-analysis where fixed-effects and random-effects modelling differed in conclusions
Supplementary Methods	Literature search strategy
Supplementary Fig. S1	Flow diagram summarizing the study selection processes

Supplementary Fig. S2	Point risk estimates of coronary heart disease and ischemic stroke for high versus low total intake of dairy products or trend
Supplementary Fig. S3	Point risk estimates of coronary heart disease and ischemic stroke for high versus low intake of low-fat dairy products or trend
Supplementary Fig. S4	Point risk estimates of coronary heart disease and ischemic stroke for high versus low intake of high-fat dairy products or trend
Supplementary Fig. S5	High versus low meta-analysis. Summary risk ratio of coronary heart disease and ischemic stroke for high versus low intake of milk
Supplementary Fig. S6	High versus low meta-analysis. Summary risk ratio of coronary heart disease and ischemic stroke for high versus low intake of yogurt
Supplementary Fig. S7	High versus low meta-analysis. Summary risk ratio of coronary heart disease and ischemic stroke for high versus low intake of cheese
Supplementary Fig. S8	High versus low meta-analysis. Summary risk ratio of coronary heart disease for high versus low intake of butter
Supplementary Fig. S9	Low risk of bias sensitivity analysis
Supplementary Fig. S10	Assessment of publication bias

Section/topic	#	Checklist item	Reported on page #
**TITLE**			
Title	1	Identify the report as a systematic review, meta-analysis, or both.	1
**ABSTRACT**			
Structured summary	2	Provide a structured summary including, as applicable: background; objectives; data sources; study eligibility criteria, participants, and interventions; study appraisal and synthesis methods; results; limitations; conclusions and implications of key findings; systematic review registration number.	3
**INTRODUCTION**			
Rationale	3	Describe the rationale for the review in the context of what is already known.	4
Objectives	4	Provide an explicit statement of questions being addressed with reference to participants, interventions, comparisons, outcomes, and study design (PICOS).	4
**METHODS**			
Protocol and registration	5	Indicate if a review protocol exists, if and where it can be accessed (e.g., Web address), and, if available, provide registration information including registration number.	5
Eligibility criteria	6	Specify study characteristics (e.g., PICOS, length of follow-up) and report characteristics (e.g., years considered, language, publication status) used as criteria for eligibility, giving rationale.	5
Information sources	7	Describe all information sources (e.g., databases with dates of coverage, contact with study authors to identify additional studies) in the search and date last searched.	5
Search	8	Present full electronic search strategy for at least one database, including any limits used, such that it could be repeated.	Supplementary Methods
Study selection	9	State the process for selecting studies (i.e., screening, eligibility, included in systematic review, and, if applicable, included in the meta-analysis).	5-6, Supplementary Fig. S1

Section/topic	#	Checklist item	Reported on page #
Data collection process	10	Describe method of data extraction from reports (e.g., piloted forms, independently, in duplicate) and any processes for obtaining and confirming data from investigators.	6
Data items	11	List and define all variables for which data were sought (e.g., PICOS, funding sources) and any assumptions and simplifications made.	6
Risk of bias in individual studies	12	Describe methods used for assessing risk of bias of individual studies (including specification of whether this was done at the study or outcome level), and how this information is to be used in any data synthesis.	6, 8, Supplementary Table S4
Summary measures	13	State the principal summary measures (e.g., risk ratio, difference in means).	7
Synthesis of results	14	Describe the methods of handling data and combining results of studies, if done, including measures of consistency (e.g., I^2) for each meta-analysis.	6-8
Risk of bias across studies	15	Specify any assessment of risk of bias that may affect the cumulative evidence (e.g., publication bias, selective reporting within studies).	8
Additional analyses	16	Describe methods of additional analyses (e.g., sensitivity or subgroup analyses, meta-regression), if done, indicating which were pre-specified.	8
**RESULTS**			
Study selection	17	Give numbers of studies screened, assessed for eligibility, and included in the review, with reasons for exclusions at each stage, ideally with a flow diagram.	9, Supplementary Fig. S1, Supplementary Table S3
Study characteristics	18	For each study, present characteristics for which data were extracted (e.g., study size, PICOS, follow-up period) and provide the citations.	9, Tables 1a and 1b, Supplementary Table S5, Supplementary Table S7
Risk of bias within studies	19	Present data on risk of bias of each study and, if available, any outcome-level assessment (see Item 12).	9, 13, Supplementary Table S4
Results of individual studies	20	For all outcomes considered (benefits or harms), present, for each study: (a) simple summary data for each intervention group and (b) effect estimates and confidence intervals, ideally with a forest plot.	Fig. 1-5, Supplementary Fig. S2-S4, Supplementary Fig. S5-S8

Section/topic	#	Checklist item	Reported on page #
Synthesis of results	21	Present results of each meta-analysis done, including confidence intervals and measures of consistency.	10-13, Tables 2a and 2b, Fig. 1-5, Supplementary Fig. S5-S8
Risk of bias across studies	22	Present results of any assessment of risk of bias across studies (see Item 15).	13, Supplementary Fig. S10
Additional analysis	23	Give results of additional analyses, if done (e.g., sensitivity or subgroup analyses, meta-regression) (see Item 16).	10-13, Supplementary Table S8-S15, Supplementary Table S16, Supplementary Fig. S9
**DISCUSSION**			
Summary of evidence	24	Summarize the main findings including the strength of evidence for each main outcome; consider their relevance to key groups (e.g., health care providers, users, and policy makers).	14
Limitations	25	Discuss limitations at study and outcome level (e.g., risk of bias), and at review level (e.g., incomplete retrieval of identified research, reporting bias).	15
Conclusions	26	Provide a general interpretation of the results in the context of other evidence, and implications for future research.	16
**FUNDING**			
Funding	27	Describe sources of funding for the systematic review and other support (e.g., supply of data); role of funders for the systematic review.	23

Supplementary Table S1. PRISMA checklist. Adapted from Liberati et al.[1].

	Europe[a]	North America[b]
Milk	200 grams	244 grams
Yogurt[c]	200 grams	244 grams
Cheese	20 grams	43 grams
Butter	-	14 grams

Supplementary Table S2. Conversion of 1 serving size to grams. [a]From Ygil 2013[2]. [b]From Institute of Medicine 1990[3]. [c]Yogurt defined as yogurt products/other soured milk products.

Reference	Reason for exclusion
van der Vijver 1992[1]; Hu 2000[2]; Kant 2000[3]; Osler 2002[4]; Sauvaget 2003[5]; Umesawa 2006[6]; Drogan 2007[7]; Shimazu 2007[8]; Brunner 2008[9]; Fung 2008[10]; Umesawa 2008[11]; Holmberg 2009[12]; Kant 2009[13]; Bernstein 2012[14]; Hansen-Krone 2012[15]; Hoevenaar-Blom 2012[16]; Li 2012[17]; Menotti 2012[18]; Misirli 2012[19]; Chan 2013[20]; Chen 2013[21]; Maruyama 2013[22]; Streppel 2014[23]; Hoşcan 2015[24]; Tektonidis 2015[25]; Chen 2016[26]; Larsson 2016[27]; Praagman 2016[28]; Mertens 2017[29]; Nanri 2017[30]; Ozawa 2017[31]; Jones 2018[32]; Asadi 2019[33]; Seah 2019[34]; Vissers 2019[35]	Exposure
Fraser 1992[36]; Mann 1997[37]; Panagiotakos 2009[38]; Bonthuis 2010[39]; de Oliveira Otto 2012[40]; Eguchi 2012[41]; Pan 2012[42]; van Aerde 2013[43]; von Ruesten 2013[44]; Huang 2014[45]; Michaëlsson 2014[46]; Prinelli 2015[47]; Wang 2015[48]; Yu 2015[49]; Um 2017[50]; Buziau 2019[51]; Fresán 2019[52]; van den Brandt 2019[53]; Virtanen 2019[54]	Outcome
Bilenko 2005[55]	Study design

Supplementary Table S3. Full-text articles excluded, with reasons.

References

1. van der Vijver, L. P. L. *et al.* Calcium intake and 28-year cardiovascular and coronary heart disease mortality in Dutch civil servants. *Int. J. Epidemiol.* **21**, 36–39 (1992).
2. Hu, F. B. *et al.* Prospective study of major dietary patterns and risk of coronary heart disease in men. *Am. J. Clin. Nutr.* **72**, 912–921 (2000).
3. Kant, A. K., Schatzkin, A., Graubard, B. I. & Schairer, C. A prospective study of diet quality and mortality in women. *JAMA* **283**, 2109–2115 (2000).
4. Osler, M. *et al.* Food intake patterns and risk of coronary heart disease: a prospective cohort study examining the use of traditional scoring techniques. *Eur. J. Clin. Nutr.* **56**, 568–574 (2002).
5. Sauvaget, C., Nagano, J., Allen, N., Grant, E. J. & Beral, V. Intake of animal products and stroke mortality in the Hiroshima/Nagasaki Life Span Study. *Int. J. Epidemiol.* **32**, 536–543 (2003).
6. Umesawa, M. *et al.* Dietary intake of calcium in relation to mortality from cardiovascular disease: The JACC Study. *Stroke* **37**, 20–26 (2006).
7. Drogan, D. *et al.* A food pattern predicting prospective weight change is associated with risk of fatal but not with nonfatal cardiovascular disease. *J. Nutr.* **137**, 1961–1967 (2007).
8. Shimazu, T. *et al.* Dietary patterns and cardiovascular disease mortality in Japan: a prospective cohort study. *Int. J. Epidemiol.* **36**, 600–609 (2007).

9. Brunner, E. J. *et al.* Dietary patterns and 15-y risks of major coronary events, diabetes, and mortality. *Am. J. Clin. Nutr.* **87**, 1414–1421 (2008).

10. Fung, T. T. *et al.* Adherence to a DASH-style diet and risk of coronary heart disease and stroke in women. *Arch. Intern. Med.* **168**, 713–720 (2008).

11. Umesawa, M. *et al.* Dietary calcium intake and risks of stroke, its subtypes, and coronary heart disease in Japanese: the JPHC Study Cohort I. *Stroke* **39**, 2449–2456 (2008).

12. Holmberg, S., Thelin, A. & Stiernström, E.-L. Food choices and coronary heart disease: a population based cohort study of rural Swedish men with 12 years of follow-up. *Int. J. Environ. Res. Public Health* **6**, 2626–2638 (2009).

13. Kant, A. K., Leitzmann, M. F., Park, Y., Hollenbeck, A. & Schatzkin, A. Patterns of recommended dietary behaviors predict subsequent risk of mortality in a large cohort of men and women in the United States. *J. Nutr.* **139**, 1374–1380 (2009).

14. Bernstein, A. M., de Koning, L., Flint, A. J., Rexrode, K. M. & Willet, W. C. Soda consumption and the risk of stroke in men and women. *Am. J. Clin. Nutr.* **95**, 1190–1199 (2012).

15. Hansen-Krone, I. J., Enga, K. F., Njølstad, I., Hansen, J. B. & Braekkan, S. K. Heart healthy diet and risk of myocardial infarction and venous thromboembolism. The Tromsø Study. *Thromb. Haemost.* **108**, 554–560 (2012).

16. Hoevenaar-Blom, M. P. *et al.* Mediterranean style diet and 12-year incidence of cardiovascular diseases: the EPIC-NL cohort study. *PLoS One* **7**, e45458 (2012).

17. Li, K., Kaaks, R., Linseisen, J. & Rohrmann, S. Associations of dietary calcium intake and calcium supplementation with myocardial infarction and stroke risk and overall cardiovascular mortality in the Heidelberg cohort of the European Prospective Investigation into Cancer and Nutrition study (EPIC-Heidelberg). *Heart* **98**, 920–925 (2012).

18. Menotti, A., Alberti-Fidanza, A., Fidanza, F., Lanti, M. & Fruttini, D. Factor analysis in the identification of dietary patterns and their predictive role in morbid and fatal events. *Public Health Nutr.* **15**, 1232–1239 (2012).

19. Misirli, G. *et al.* Relation of the traditional Mediterranean diet to cerebrovascular disease in a Mediterranean population. *Am. J. Epidemiol.* **176**, 1185–1192 (2012).

20. Chan, R., Chan, D. & Woo, J. The association of a priori and a posterior dietary patterns with the risk of incident stroke in chinese older people in Hong Kong. *J. Nutr. Heal. Aging* **17**, 866–874 (2013).

21. Chen, Y. *et al.* Prospective investigation of major dietary patterns and risk of cardiovascular mortality in Bangladesh. *Int. J. Cardiol.* **167**, 1495–1501 (2013).

22. Maruyama, K. *et al.* Dietary patterns and risk of cardiovascular deaths among middle-aged Japanese: JACC Study. *Nutr. Metab. Cardiovasc. Dis.* **23**, 519–527 (2013).

23. Streppel, M. T. *et al.* Nutrient-rich foods, cardiovascular diseases and all-cause mortality: the Rotterdam study. *Eur. J. Clin. Nutr.* **68**, 741–747 (2014).

24. Hoşcan, Y., Yiğit, F. & Müderrisoğlu, H. Adherence to Mediterranean diet and its relation with cardiovascular diseases in Turkish population. *Int. J. Clin. Exp. Med.* **8**, 2860–2866 (2015).

25. Tektonidis, T. G., Åkesson, A., Gigante, B., Wolk, A. & Larsson, S. C. A Mediterranean diet and risk of myocardial infarction, heart failure and stroke: a population-based cohort study. *Atherosclerosis* **243**, 93–98 (2015).

26. Chen, M. *et al.* Dairy fat and risk of cardiovascular disease in 3 cohorts of US adults. *Am. J. Clin. Nutr.* **104**, 1209–1217 (2016).

27. Larsson, S. C., Wallin, A. & Wolk, A. Dietary Approaches to Stop Hypertension diet and incidence of stroke. Results from 2 prospective cohorts. *Stroke* **47**, 986–990 (2016).

28. Praagman, J. *et al.* The association between dietary saturated fatty acids and ischemic heart disease depends

on the type and source of fatty acid in the European Prospective Investigation into Cancer and Nutrition-Netherlands cohort. *Am. J. Clin. Nutr.* **103**, 356–365 (2016).

29. Mertens, E., Markey, O., Geleijnse, J. M., Givens, D. I. & Lovegrove, J. A. Dietary patterns in relation to cardiovascular disease incidence and risk markers in a middle-aged British male population: data from the Caerphilly Prospective Study. *Nutrients* **9**, 75 (2017).

30. Nanri, A. *et al.* Dietary patterns and all-cause, cancer, and cardiovascular disease mortality in Japanese men and women: the Japan public health center-based prospective study. *PLoS One* **12**, e0174848 (2017).

31. Ozawa, M. *et al.* Dietary protein intake and stroke risk in a general Japanese population: the Hisayama Study. *Stroke* **48**, 1478–1486 (2017).

32. Jones, N. R. V., Forouhi, N. G., Khaw, K. T., Wareham, N. J. & Monsivais, P. Accordance to the Dietary Approaches to Stop Hypertension diet pattern and cardiovascular disease in a British, population-based cohort. *Eur. J. Epidemiol.* **33**, 235–244 (2018).

33. Asadi, Z. *et al.* Association of dietary patterns and risk of cardiovascular disease events in the MASHAD cohort study. *J. Hum. Nutr. Diet.* **32**, 789–801 (2019).

34. Seah, J. Y. H., Ong, C. N., Koh, W.-P., Yuan, J.-M. & van Dam, R. M. A dietary pattern derived from reduced rank regression and fatty acid biomarkers is associated with lower risk of type 2 diabetes and coronary artery disease in Chinese adults. *J. Nutr.* **149**, 2001–2010 (2019).

35. Vissers, L. E. T. *et al.* Fatty acids from dairy and meat and their association with risk of coronary heart disease. *Eur. J. Nutr.* **58**, 2639–2647 (2019).

36. Fraser, G. E., Sabaté, J., Beeson, W. L. & Strahan, T. M. A possible protective effect of nut consumption on risk of coronary heart disease. The Adventist Health Study. *Arch. Intern. Med.* **152**, 1416–1424 (1992).

37. Mann, J. I., Appleby, P. N., Key, T. J. & Thorogood, M. Dietary determinants of ischaemic heart disease in health conscious individuals. *Heart* **78**, 450–455 (1997).

38. Panagiotakos, D. *et al.* Dietary patterns and 5-year incidence of cardiovascular disease: a multivariate analysis of the ATTICA study. *Nutr. Metab. Cardiovasc. Dis.* **19**, 253–263 (2009).

39. Bonthuis, M., Hughes, M. C. B., Ibiebele, T. I., Green, A. C. & van der Pols, J. C. Dairy consumption and patterns of mortality of Australian adults. *Eur. J. Clin. Nutr.* **64**, 569–577 (2010).

40. de Oliveira Otto, M. C. *et al.* Dietary intake of saturated fat by food source and incident cardiovascular disease: the Multi-Ethnic Study of Atherosclerosis. *Am. J. Clin. Nutr.* **96**, 397–404 (2012).

41. Eguchi, E. *et al.* Healthy lifestyle behaviours and cardiovascular mortality among Japanese men and women: the Japan collaborative cohort study. *Eur. Heart J.* **33**, 467–477 (2012).

42. Pan, A. *et al.* Red meat consumption and mortality. Results from 2 prospective cohort studies. *Arch. Intern. Med.* **172**, 555–563 (2012).

43. van Aerde, M. A. *et al.* Dairy intake in relation to cardiovascular disease mortality and all-cause mortality: the Hoorn Study. *Eur. J. Nutr.* **52**, 609–616 (2013).

44. von Ruesten, A., Feller, S., Bergmann, M. M. & Boeing, H. Diet and risk of chronic diseases: results from the first 8 years of follow-up in the EPIC-Potsdam study. *Eur. J. Clin. Nutr.* **67**, 412–419 (2013).

45. Huang, L. Y., Wahlqvist, M. L., Huang, Y. C. & Lee, M. S. Optimal dairy intake is predicated on total, cardiovascular, and stroke mortalities in a Taiwanese cohort. *J. Am. Coll. Nutr.* **33**, 426–436 (2014).

46. Michaëlsson, K. *et al.* Milk intake and risk of mortality and fractures in women and men: cohort studies. *BMJ* **349**, g6015 (2014).

47. Prinelli, F. *et al.* Mediterranean diet and other lifestyle factors in relation to 20-year all-cause mortality: a cohort study in an Italian population. *Br. J. Nutr.* **113**, 1003–1011 (2015).

48. Wang, C., Yatsuya, H., Tamakoshi, K., Iso, H. & Tamakoshi, A. Milk drinking and mortality: findings from the Japan Collaborative Cohort Study. *J. Epidemiol.* **25**, 66–73 (2015).

49. Yu, D. *et al.* Healthy eating and risks of total and cause-specific death among low-income populations of African-Americans and other adults in the Southeastern United States: a prospective cohort study. *PLoS Med.* **12**, e1001830 (2015).

50. Um, C. Y., Judd, S. E., Flanders, W. D., Fedirko, V. & Bostick, R. M. Associations of calcium and dairy products with all-cause and cause-specific mortality in the REasons for Geographic and Racial Differences in Stroke (REGARDS) prospective cohort study. *Nutr. Cancer* **69**, 1185–1195 (2017).

51. Buziau, A. M., Soedamah-Muthu, S. S., Geleijnse, J. M. & Mishra, G. D. Total fermented dairy food intake is inversely associated with cardiovascular disease risk in women. *J. Nutr.* **149**, 1797–1804 (2019).

52. Fresán, U. *et al.* Adherence to the 2015 Dietary Guidelines for Americans and mortality risk in a Mediterranean cohort: The SUN project. *Prev. Med.* **118**, 317–324 (2019).

53. van den Brandt, P. A. Red meat, processed meat, and other dietary protein sources and risk of overall and cause-specific mortality in The Netherlands Cohort Study. *Eur. J. Epidemiol.* **34**, 351–369 (2019).

54. Virtanen, H. E. K. *et al.* Dietary proteins and protein sources and risk of death: the Kuopio Ischaemic Heart Disease Risk Factor Study. *Am. J. Clin. Nutr.* **109**, 1462–1471 (2019).

55. Bilenko, N., Fraser, D., Vardi, H., Shai, I. & Shahar, D. R. Mediterranean diet and cardiovascular diseases in an Israeli population. *Prev. Med.* **40**, 299–305 (2005).

	Selection		Exposure		Outcome		Comparability	
First author's last name and publication year	Demonstration that outcome was not present at enrollment	Participants lost to follow-up unlikely to have affected the results (≤10% lost)	Information on food intake in participants collected through multiple replicates of 24-h recalls or 1-day food records, or collected through a semiquantitative FFQ or a dietary history interview	Method for collecting information on food intake of participants validated	Ascertainment of outcome through secure sources (e.g. medical records or record linkage of the study population with a register providing CHD diagnoses)	Ascertainment of outcome blinded to food intake	Adjustment for gender (as appropriate) and age	Adjustment for additional risk factors of CHD
Hu 1999	+	?	+	+	+	+	+	+
Al-Delaimy 2003	+	?	+	+	+	?	+	+
Elwood 2004	?	+	+	+	+	?	+	+
Buckland. 2009	+	?	+	+	+	+	+	+
Martínez-González 2011	+	?	+	+	+	+	+	+
Sonestedt 2011	+	+	+	+	+	+	+	+
Dilis 2012	+	?	+	+	+	?	+	+
Avalos 2013	+	+	+	+	-	?	+	+
Dalmeijer 2013	+	?	+	+	+	+	+	+
Patterson 2013	+	+	+	+	+	+	+	+
Soedamah-Muthu 2013	+	+	+	+	+	?	-	+
Haring 2014	+	?	+	+	+	?	+	+
Bergholdt 2015	+	+	+	?	+	+	+	+
Praagman 2015	+	?	+	+	+	?	+	+
Liu 2017	+	+	+	+	+	?	+	+
Dehghan 2018	+	?	-	+	?	?	+	+
Koskinen 2018	+	+	+	?	+	+	+	+
Johansson 2019	-	?	+	+	+	+	+	+
Key 2019	+	+	+	+	+	?	+	+
Talaei 2019	+	-	-	+	+	?	+	+

Supplementary Table S4a. Risk of bias judgements of included studies on intake of dairy products and risk of CHD based on the Newcastle-Ottawa scale (NOS) for assessing the quality of cohort studies in meta-analysis[4]. CHD, indicates coronary heart disease; FFQ, food frequency questionnaire. +, indicates yes (equals "low risk" of bias); -, no (equals "high-risk" of bias); ?, unknown (equals "unclear risk" of bias).

First author's last name and publication year	Selection		Exposure		Outcome		Comparability	
	Demonstration that outcome was not present at enrollment	Participants lost to follow-up unlikely to have affected the results (≤10% lost)	Information on food intake in participants collected through multiple replicates of 24-h recalls or 1-day food records, or collected through a semiquantitative FFQ or a dietary history interview	Method for collecting information on food intake of participants validated	Ascertainment of outcome through secure sources (e.g. medical records or record linkage of the study population with a register providing ischemic stroke diagnoses and validation of the diagnoses)	Ascertainment of outcome blinded to food intake	Adjustment for gender (as appropriate) and age	Adjustment for additional risk factors of ischemic stroke
Abbott 1996	+	?	-	+	+	?	-	-
Iso 1999	+	+	+	+	+	+	+	+
Elwood 2004	?	+	+	+	+	?	+	+
Larsson 2009	+	?	+	+	+	+	+	+
Bernstein 2012	+	?	+	+	+	?	+	+
Larsson 2012	+	+	+	+	-	+	+	+
Yaemsiri 2012	+	+	+	+	+	?	+	+
Lin 2013	+	?	+	+	?	?	+	+
Haring 2015	+	?	+	+	+	?	+	+
Liu 2017	+	+	+	+	+	?	+	+
Laursen 2018	+	+	+	+	+	+	+	+
Laursen 2019	+	?	+	+	-	+	-	+

Supplementary Table S4b. Risk of bias judgements of included studies on intake of dairy products and risk of ischemic stroke based on the Newcastle-Ottawa scale (NOS) for assessing the quality of cohort studies in meta-analysis[4]. FFQ, indicates food frequency questionnaire. +, indicates yes (equals "low risk" of bias); -, no (equals "high-risk" of bias); ?, unknown (equals "unclear risk" of bias).

First author's last name and publication year	Exposure	Exposure definition	Exposure in data synthesis[a]
Hu 1999	Low-fat dairy	Skim milk, low-fat milk, yogurt and cottage cheese	Low-fat dairy products
	High-fat dairy	Whole milk, hard or cream cheese, ice cream and butter	High-fat dairy products
	Skim milk	-	Low-fat milk
	Whole milk	-	High-fat milk
Al-Delaimy 2003	Dairy	-	Total dairy products
Elwood 2004	Milk	-	Milk
Buckland 2009	Dairy products	Low-fat and high-fat milk, yogurt, cheese, cream desserts, and dairy and nondairy creams	Total dairy products
Martínez-González 2011	Dairy	-	Total dairy products
Sonestedt 2011	Total dairy	Milk, cheese (10% fat), cream and butter (including the milk-based spread Bregott)	Total dairy products
	Butter	-	Butter
Dilis 2012	Dairy foods	-	Total dairy products
Avalos 2013	Non-fat milk	-	Low-fat milk
	Whole milk	-	High-fat milk
	Yogurt	-	Yogurt
	Cheese	-	Cheese
	Low-fat cheese	-	Low-fat cheese
	Butter	-	Butter
Dalmeijer 2013	Total dairy	All dairy food products, except for butter and ice cream	Total dairy products
	Low-fat dairy	Milk and milk products with <2 g/100 g fat (skimmed or semi-skimmed milk products) and cheese with <20 g/100 g fat	Low-fat dairy products
	High-fat dairy	Milk and milk products with ≥2 g/100 g fat (whole milk products) and cheese with ≥20 g/100 g fat	High-fat dairy products
Patterson 2013	Total dairy foods	Milk, cultured milk and yogurt, cheese, cream and crème fraîche	Total dairy products
	Milk	Full-fat (≥3.0% fat), semiskimmed (≤1.5% fat), skimmed (0.5% fat) and milk in pancakes	Milk
	Low-fat milk	Skimmed (0.5% fat) and semiskimmed (≤1.5% fat)	Low-fat milk
	Full-fat milk	≥3.0% fat	High-fat milk
	Cultured milk and yogurt	Full-fat (≥3.0% fat) and low-fat (≤1.5% fat)	Yogurt
	Low-fat cultured milk and yogurt	≤1.5% fat	Low-fat yogurt
	Full-fat cultured milk and yogurt	≥3.0% fat	High-fat yogurt
	Cheese	Full-fat (>17% fat), low-fat (≤17% fat) and cottage cheese and quark	Cheese
	Low-fat cheese	Low-fat varieties (10-17%)	Low-fat cheese
	Full-fat cheese	Full-fat (>17% fat)	High-fat cheese
Soedamah-Muthu 2013	Total dairy	All dairy products, except for butter and ice cream	Total dairy products
	Low-fat dairy	Cottage cheese, semi-skimmed, skimmed milk and milk-based hot drinks	Low-fat dairy products

First author's last name and publication year	Exposure	Exposure definition	Exposure in data synthesis[a]
	High-fat dairy	Full-fat cheese, yogurt, milk puddings and whole and Channel Islands milk	High-fat dairy products
	Total milk	Whole-fat and low-fat	Milk
	Yogurt	-	Yogurt
	Cheese	-	Cheese
Haring 2014	Dairy	-	Total dairy products
	Low-fat dairy	-	Low-fat dairy products
	High-fat dairy	-	High-fat dairy products
	Low-fat dairy *for* high fat dairy	-	Low-fat dairy products *for* high-fat dairy products
Bergholdt 2015	Milk	Whole milk, semi-skimmed milk and skimmed milk	Milk
Praagman 2015	Total dairy	Milk, buttermilk, yogurt, coffee creamer, curd, pudding, porridge, custard, whipped cream, ice cream and cheese	Total dairy products
	Low-fat dairy	Milk and milk products with a fat content <2/100 g and cheese with a fat content <20/100 g	Low-fat dairy products
	High-fat dairy	Milk and milk products with a fat content ≥2/100 g and cheese with a fat content ≥20/100 g	High-fat dairy products
	Yogurt	-	Yogurt
	Cheese	All types of cheese, except for curd	Cheese
Liu 2017	Butter	-	Butter
Dehghan 2018	Total dairy	Milk, yoghurt, various types of cheese and yoghurt drink	Total dairy products
Koskinen 2018	Total dairy products	Fermented and non-fermented dairy products	Total dairy products
	Cheese	All fermented cheese products like Blue, Edam, Gouda and Swiss cheese	Cheese
	Low-fat milk	Semi-skimmed milk and skimmed milk (≤1.9% fat)	Low-fat milk
	High-fat milk	Farm milk, whole milk and cream milk	High-fat milk
	Butter	-	Butter
Johansson 2019	Non-fermented milk	0.5%, 1.5% and 3.0% fat	Milk
	Low-fat non-fermented milk	-	Low-fat milk
	High-fat non-fermented milk	-	High-fat milk
	Fermented milk	0.5 and ≥3% fat	Yogurt
	Low-fat fermented milk	0.5% fat	Low-fat yogurt
	High-fat fermented milk	≥3% fat	High-fat yogurt
	Cheese	10-15% fat and ≥28% fat	Cheese
	Low-fat cheese	10-15% fat	Low-fat cheese
	High-fat cheese	≥28% fat	High-fat cheese
	Butter	Pure butter and a mixed spread with 70% butter	Butter
Key 2019	Milk	Plain milk including buttermilk, excluding milk-based beverages	Milk
	Yogurt	-	Yogurt
	Cheese	-	Cheese
Talaei 2019	Whole milk	-	High-fat milk

Supplementary Table S5a. Definition of dairy products as described in included studies on intake of dairy products and risk of coronary heart disease. [a]Yogurt defined as yogurt/other soured milk products.

First author's last name and publication year	Exposure	Exposure definition	Exposure in data synthesis[a]
Abbott 1996	Milk	-	Milk
Iso 1999	Milk	-	Milk
	Yogurt	-	Yogurt
	Hard cheese	-	Cheese
Elwood 2004	Milk	-	Milk
Larsson 2009	Total dairy	-	Total dairy products
	Low-fat milk	-	Low-fat milk
	Whole milk	-	High-fat milk
	Yogurt	-	Yogurt
	Cheese	-	Cheese
	Butter	-	Butter
Bernstein 2012	Low-fat dairy	Skim milk (1%), low-fat milk (2%), yogurt, cottage and ricotta cheeses, low-fat cheese and sherbet	Low-fat dairy products
	Whole-fat dairy	Whole milk, ice cream, hard cheese, full-fat cheese, cream, sour cream, cream cheese and butter	High-fat dairy products
Larsson 2012	Total dairy	Low-fat milk (0.5% fat), medium-fat milk (1.5% fat), full-fat milk (3% fat), milk in pancakes, low-fat sour milk and yogurt (0.5% fat), full-fat sour milk and yogurt (3% fat), cottage cheese (4% fat), low-fat cheese (10%-17% fat), full-fat cheese (28% fat), ice cream, cream and crème fraîche	Total dairy products
	Low-fat dairy	Low-fat milk (0.5% fat), medium-fat milk (1.5% fat), low-fat sour milk and yogurt (0.5% fat), cottage cheese (4% fat) and low-fat cheese (10%-17% fat)	Low-fat dairy products
	Full-fat dairy	Full-fat milk (3% fat), full-fat sour milk and yogurt (3% fat), full-fat cheese (28% fat), ice cream, cream and crème fraîche	High-fat dairy products
	Milk	Low-fat milk (0.5% fat), medium-fat milk (1.5% fat), full-fat milk (3% fat) and milk in pancakes	Milk
	Sour milk and yogurt	Low-fat sour milk and yogurt (0.5% fat) and full-fat sour milk and yogurt (3% fat)	Yogurt
	Cheese	Low-fat cheese (10%-17% fat) and full-fat cheese (28% fat)	Cheese
Yaemsiri 2012	Dairy	-	Total dairy products
Lin 2013	Dairy	Milk of any kind, cheese and yogurt	Total dairy products
Haring 2015	Dairy	-	Total dairy products
	Low-fat dairy	-	Low-fat dairy products
	High-fat dairy	-	High-fat dairy products
Liu 2017	Butter	-	Butter
Laursen 2018	Low-fat milk *for* whole-fat milk	Skimmed milk (0.3% fat) and semi-skimmed milk (1.5% fat) *for* whole-fat milk (3.5% fat)	Low-fat milk *for* high-fat milk
	Semi-skimmed fermented milk *for* whole-fat fermented milk	Yoghurt-type products (1.5% fat) *for* yoghurt-type products (3.5% fat)	Low-fat yogurt *for* high-fat yogurt
	Semi-skimmed fermented milk *for* low-fat milk	Yoghurt-type products (1.5% fat) *for* skimmed milk (0.3% fat) and semi-skimmed milk (1.5% fat)	Low-fat yogurt *for* low-fat milk
	Semi-skimmed fermented milk *for* whole-fat milk	Yoghurt-type products (1.5% fat) *for* whole-fat milk (3.5% fat)	Low-fat yogurt *for* high-fat milk
	Whole-fat fermented milk *for* low-fat milk	Yoghurt-type products (3.5% fat) *for* skimmed milk (0.3% fat) and semi-skimmed milk (1.5% fat)	High-fat yogurt *for* low-fat milk

First author's last name and publication year	Exposure	Exposure definition	Exposure in data synthesis[a]
	Whole-fat fermented milk *for* whole-fat milk	Yoghurt-type products (3.5% fat) *for* whole-fat milk (3.5% fat)	High-fat yogurt *for* high-fat milk
	Cheese *for* low-fat milk	Semi-hard cheese, soft matured cheese, blue cheese, cottage cheese and cream cheese *for* skimmed milk (0.3% fat) and semi-skimmed milk (1.5% fat)	Cheese *for* low-fat milk
	Cheese *for* whole-fat milk	Semi-hard cheese, soft matured cheese, blue cheese, cottage cheese and cream cheese *for* whole-fat milk (3.5% fat)	Cheese *for* high-fat milk
	Cheese *for* semi-skimmed fermented milk	Semi-hard cheese, soft matured cheese, blue cheese, cottage cheese and cream cheese *for* yoghurt-type products (1.5% fat)	Cheese *for* low-fat yogurt
	Cheese *for* whole-fat fermented milk	Semi-hard cheese, soft matured cheese, blue cheese, cottage cheese and cream cheese for yoghurt-type products (3.5% fat)	Cheese *for* high-fat yogurt
	Cheese *for* butter	Semi-hard cheese, soft matured cheese, blue cheese, cottage cheese and cream cheese *for* butter	Cheese *for* butter
Laursen 2019	Low-fat milk *for* whole-fat milk	Skimmed and semi-skimmed milk (<2% fat) *for* whole-fat, raw and powdered milk (≥3% fat)	Low-fat milk *for* high-fat milk
	Low-fat milk *for* butter	Skimmed and semi-skimmed milk (<2% fat) *for* butter	Low-fat milk *for* butter
	Whole-fat milk *for* butter	Whole-fat, raw and powdered milk (≥3%) fat *for* butter	High-fat milk *for* butter
	Low-fat yogurt *for* whole-fat yogurt	Skimmed and semi-skimmed regular and drink yogurt (<2% fat), curd *for* regular and drink yogurt (≥3% fat)	Low-fat yogurt *for* high-fat yogurt
	Low-fat yogurt *for* low-fat milk	Skimmed and semi-skimmed regular and drink yogurt (<2% fat), curd *for* skimmed and semi-skimmed milk (<2% fat)	Low-fat yogurt *for* low-fat milk
	Low-fat yogurt *for* whole-fat milk	Skimmed and semi-skimmed regular and drink yogurt (<2% fat), curd *for* whole-fat, raw and powdered milk (≥3% fat)	Low-fat yogurt *for* high-fat milk
	Low-fat yogurt *for* butter	Skimmed and semi-skimmed regular and drink yogurt (<2% fat), curd *for* butter	Low-fat yogurt *for* butter
	Whole-fat yogurt *for* low-fat milk	Regular and drink yogurt (≥3% fat) *for* skimmed and semi-skimmed milk (<2% fat)	High-fat yogurt *for* low-fat milk
	Whole-fat yogurt *for* whole-fat milk	Regular and drink yogurt (≥3% fat) *for* whole-fat, raw and powdered milk (≥3% fat)	High-fat yogurt *for* high-fat milk
	Whole-fat yogurt *for* butter	Regular and drink yogurt (≥3%) fat *for* butter	High-fat yogurt *for* butter
	Low-fat milk *for* cheese	Skimmed and semi-skimmed milk (<2%) fat *for* cheese	Cheese *for* low-fat milk
	Whole-fat milk *for* cheese	Whole-fat, raw and powdered milk (≥3% fat) *for* cheese	Cheese *for* high-fat milk
	Low-fat yogurt *for* cheese	Skimmed and semi-skimmed regular and drink yogurt (<2% fat), curd *for* cheese	Cheese *for* low-fat yogurt
	Whole-fat yogurt *for* cheese	Regular and drink yogurt (≥3%) fat *for* cheese	Cheese *for* high-fat yogurt
	Cheese *for* butter	-	Cheese *for* butter

Supplementary Table S5b. Definition of dairy products as described in included studies on intake of dairy products and risk of ischemic stroke. [a]Yogurt defined as yogurt/other soured milk products.

Exposure[a]	HL	HL_subgender	HL_subcontinent	DRlin	DRlin_subgender	DRlin_subcontinent	DRnonlin
Milk	n = 6[5–10]	n = 3[5,6,9]	-	n = 5[5–8,10]	n = 3[5,6,10]	-	n = 4[5–8]
Low-fat milk	n = 5[6,9,11–13]	n = 4[6,11–13]	n = 5[6,9,11–13]	n = 3[6,12,13]	n = 3[6,12,13]	n = 3[6,12,13]	n = 2[6,13]
High-fat milk	n = 6[6,9,11–14]	n = 4[6,11–13]	n = 6[6,9,11–14]	n = 4[6,12–14]	n = 3[6,12,13]	n = 4[6,12–14]	n = 3[6,13,14]
Yogurt	n = 6[6,7,9,10,12,15]	n = 3[6,9,12]	n = 6[6,7,9,10,12,15]	n = 5[6,7,10,12,15]	n = 3[6,10,12]	n = 5[6,7,10,12,15]	n = 3[6,7,15]
Low-fat yogurt	n = 2[6,9]	-	-	-	-	-	-
High-fat yogurt	n = 2[6,9]	-	-	-	-	-	-
Cheese	n = 7[6,7,9,10,12,13,15]	n = 4[6,9,12,13]	n = 7[6,7,9,10,12,13,15]	n = 6[6,7,10,12,13,15]	n = 4[6,10,12,13]	n = 6[6,7,10,12,13,15]	n = 4[6,7,13,15]
Low-fat cheese	n = 3[6,9,12]	n = 2[6,12]	n = 3[6,9,12]	n = 2[6,12]	n = 2[6,12]	n = 2[6,12]	-
High-fat cheese	n = 2[6,9]	-	-	-	-	-	-
Butter	n = 4[9,12,13,16]	n = 3[9,12,13]	n = 4[9,12,13,16]	n = 3[12,13,17]	n = 3[12,13,17]	n = 3[12,13,17]	-

Supplementary Table S6a. Overview of which of the included studies contributed to each of the meta-analyses on intake of dairy product subgroups and risk of coronary heart disease; DRlin indicates linear dose-response meta-analysis; DRlin_subcontinent, linear dose-response meta-analysis (subgroup analysis by continent); DRlin_subgender, linear dose-response meta-analysis (subgroup analysis by gender); DRnonlin, non-linear dose-response meta-analysis; HL, high versus low meta-analysis; HL_subcontinent, high versus low meta-analysis (subgroup analysis by continent); HL_subgender, high versus low meta-analysis (subgroup analysis by gender). [a]Yogurt defined as yogurt products/other soured milk products.

Exposure	HL	HL_subgender	HL_subcontinent	DRlin	DRlin_subgender	DRlin_subcontinent	DRnonlin
Milk	n = 3[5,18,19]	n = 2[5,18]	n = 3[5,18,19]	n = 3[5,18,19]	n = 2[5,18]	n = 3[5,18,19]	n = 2[5,19]
Yogurt	n = 3[18–20]	n = 2[18,20]	n = 3[18–20]	n = 2[18,19]	-	-	-
Cheese	n = 3[18–20]	n = 2[18,20]	n = 3[18–20]	n = 3[18–20]	n = 2[18,20]	n = 3[18–20]	n = 2[19,20]
Butter	-	-	-	n = 2[17,20]	-	-	
Low-fat milk *for* high-fat milk	-	-	-	n = 2[21,22]	-	-	-
Low-fat yogurt *for* high-fat yogurt	-	-	-	n = 2[21,22]	-	-	-
Low-fat yogurt *for* low-fat milk	-	-	-	n = 2[21,22]	-	-	-
Low-fat yogurt *for* high-fat milk	-	-	-	n = 2[21,22]	-	-	-
High-fat yogurt *for* low-fat milk	-	-	-	n = 2[21,22]	-	-	-
High-fat yogurt *for* high-fat milk	-	-	-	n = 2[21,22]	-	-	-
Cheese *for* low-fat milk	-	-	-	n = 2[21,22]	-	-	-
Cheese *for* high-fat milk	-	-	-	n = 2[21,22]	-	-	-
Cheese *for* low-fat yogurt	-	-	-	n = 2[21,22]	-	-	-
Cheese *for* high-fat yogurt	-	-	-	n = 2[21,22]	-	-	-
Cheese *for* butter	-	-	-	n = 2[21,22]	-	-	-

Supplementary Table S6b. Overview of which of the included studies contributed to each of the meta-analyses on intake of dairy product subgroups and risk of ischemic stroke. DRlin, indicates linear dose-response meta-analysis; DRlin_subcontinent, linear dose-response meta-analysis (subgroup analysis by continent); DRlin_subgender, linear dose-response meta-analysis (subgroup analysis by gender); DRnonlin, non-linear dose-response meta-analysis; HL, high versus low meta-analysis; HL_subcontinent, high versus low meta-analysis (subgroup analysis by continent); HL_subgender, high versus low meta-analysis (subgroup analysis by gender). [a]Yogurt defined as yogurt products/other soured milk products.

First author's last name and publication year	Funding sources (current study)
Hu 1999	The National Institutes of Health
Al-Delaimy 2003	The National Institutes of Health
Elwood 2004	The Food Standards Agency
Buckland 2009	A scientific research grant (FIS) and the Health Research Institute Carlos III, Spain (RETIC) from the Spanish Ministry of Health
Martínez-González 2011	The official public Institutes of the Spanish Government for funding health research (Instituto de Salud Carlos III) and the Department of Health of the Navarra Regional Government
Sonestedt 2011	The Lund University Diabetes Center (LUDC), the Swedish Medical Research Council, the Swedish Heart and Lung Foundation; the Region Skåne, the Skåne University Hospital Foundation, the Albert Påhlsson Research Foundation and the Crafoord foundation
Dilis 2012	The Hellenic Health Foundation, the Stavros Niarchos Foundation and the Greek Ministry of Health
Avalos 2013	The National Institutes of Health/National Institute on Aging and the National Institute of Diabetes and Digestive and Kidney Diseases
Dalmeijer 2013	The Netherlands Heart Foundation
Patterson 2013	The Swedish Council for Working Life and Social Research and the Swedish Research Council/Infrastructure Medicine
Soedamah-Muthu 2013	The British Heart Foundation and the Stroke Association
Haring 2014	The German Research Foundation (DFG) and the University of Würtzburg
Bergholdt 2015	The Research Unit at Næstved Hospital, the Danish Dairy Research Foundation and the Regional Research Unit in Region Zealand
Praagman 2015	The Dutch Dairy Organization (NZO)
Liu 2017	-
Dehghan 2018	The Mary W Burke endowed chair of the Heart and Stroke Foundation of Ontario
Koskinen 2018	The Finnish Foundation for Cardiovascular Research, the Otto A. Malm Foundation, the Olvi Foundation, the Yrjö Jahnsson Foundation and the North Savo Regional Fund
Johansson 2019	The Swedish Research Council for Health, Working Life and Welfare (FORTE)
Key 2019	The UK Medical Research Council, Cancer Research UK and the Wellcome Trust
Talaei 2019	-

Supplementary Table S7a. Funding sources of included studies on intake of dairy products and risk of coronary heart disease.

First author’s last name and publication year	Funding sources (current study)
Abbott 1996	The National Heart, Lung, and Blood Institute
Iso 1999	The National Institutes of Health and the Japan Society for the Promotion of Science
Elwood 2004	The Food Standards Agency
Larsson 2009	The Swedish Council for Working Life and Social Research
Bernstein 2012	The National Institutes of Health, Department of Health and Human Services and the Harvard Human Nutrition Program
Larsson 2012	The Swedish Council for Working Life and Social Research (FAS), the Swedish Research Council and Karolinska Institutet
Yaemsiri 2012	The National Institute of Neurological Disorders and Stroke and the American Heart Association Mid-Atlantic Predoctoral Fellowship
Lin 2013	-
Haring 2015	-
Liu 2017	-
Laursen 2018	The Danish Council for Strategic Research (Innovation Fund Denmark)
Laursen 2019	The Unilever Research and Development

Supplementary Table S7b. Funding sources of included studies on intake of dairy products and risk of ischemic stroke.

		Number of studies	RR	95% CI	I^2 (*P* heterogeneity between studies)	*P* heterogeneity between subgroups
Milk	Total	6	1.02	0.92, 1.13	67% (<0.01)	
	Gender					0.95
	Men	2	1.01	0.60, 1.69		
	Women	2	1.02	0.83, 1.26		
Low-fat milk	Total	5	1.05	0.92, 1.20	59% (0.03)	
	Gender					0.54
	Men	2	1.14	0.93, 1.40		
	Women	3	1.02	0.77, 1.36		
	Continent					0.89
	Asia	-	-	-		
	Europe	3	1.08	0.99, 1.17		
	North America	2	1.05	0.72, 1.52		
High-fat milk	Total	6	1.16	1.01, 1.33	53% (0.04)	
	Gender					0.53
	Men	2	1.10	0.89, 1.37		
	Women	3	1.24	0.92, 1.68		
	Continent					0.47
	Asia	1	1.32	0.92, 1.90		
	Europe	3	1.07	0.97, 1.18		
	North America	2	1.21	0.84, 1.75		

Supplementary Table S8a. High versus low intake meta-analysis for milk and coronary heart disease by gender and continent. CI indicates confidence interval; RR, risk ratio.

		Number of studies	RR	95% CI	I^2 (*P* heterogeneity between studies)	*P* heterogeneity between subgroups
Milk	Total	3	0.88	0.79, 0.98	0% (0.52)	
	Gender					0.83
	Men	1	0.66	0.24, 1.81		
	Women	1	0.74	0.51, 1.07		
	Continent					0.32
	Asia	-	-	-		
	Europe	2	0.90	0.80, 1.00[a]		
	North America	1	0.74	0.51, 1.07		

Supplementary Table S8b. High versus low intake meta-analysis for milk and ischemic stroke by gender and continent. [a]*P* >0.05 for the association between milk and ischemic stroke in studies from Europe. CI indicates confidence interval; RR, risk ratio.

		Number of studies	RR	95% CI	I^2 (*P* heterogeneity between studies)	*P* heterogeneity between subgroups
Milk	Total	5	1.02	1.00, 1.04[a]	0% (0.52)	
	Gender					0.24
	Men	2	1.01	0.98, 1.05		
	Women	2	1.05	1.00, 1.09[b]		
Low-fat milk	Total	3	1.05	0.96, 1.13	24% (0.27)	
	Gender					0.48
	Men	2	1.04	0.97, 1.12		
	Women	2	1.29	0.72, 2.32		
	Continent					0.21
	Asia	-	-	-		
	Europe	2	1.03	0.97, 1.10		
	North America	1	1.43	0.87, 2.37		
High-fat milk	Total	4	1.08	1.00, 1.16[c]	0% (0.94)	
	Gender					0.80
	Men	2	1.06	0.98, 1.15		
	Women	2	1.09	0.92, 1.29		
	Continent					0.71
	Asia	1	1.20	0.90, 1.60		
	Europe	2	1.07	0.99, 1.15		
	North America	1	1.00	0.66, 1.51		

Supplementary Table S9a. Linear dose-response meta-analysis for milk intake (per 200 g/day) and coronary heart disease by gender and continent. [a]*P* = 0.12 for the association between milk and coronary heart disease; [b]*P* = 0.03 for the association between milk and coronary heart disease in studies among women; [c]*P* = 0.04 for the association between high-fat milk and coronary heart disease. CI indicates confidence interval; RR, risk ratio.

		Number of studies	RR	95% CI	I^2 (*P* heterogeneity between studies)	*P* heterogeneity between subgroups
Milk	Total	3	0.96	0.91, 1.01	16% (0.31)	
	Gender					0.94
	Men	1	0.89	0.74, 1.07		
	Women	1	0.88	0.76, 1.03		
	Continent					0.22
	Asia	-	-	-		
	Europe	2	0.97	0.94, 1.00[a]		
	North America	1	0.88	0.76, 1.03		

Supplementary Table S9b. Linear dose-response meta-analysis for milk intake (per 200 g/day) and ischemic stroke by gender and continent. [a]*P* = 0.09 for the association between milk and ischemic stroke in studies from Europe. CI indicates confidence interval; RR, risk ratio.

		Number of studies	RR	95% CI	I^2 (*P* heterogeneity between studies)	*P* heterogeneity between subgroups
Yogurt	Total	6	0.99	0.91, 1.08	49% (0.06)	
	Gender					0.97
	Men	2	1.00	0.79, 1.27		
	Women	3	0.99	0.84, 1.17		
	Continent					<0.05
	Asia	-	-	-		
	Europe	5	0.96	0.89, 1.04		
	North America	1	1.25	0.97, 1.61		

Supplementary Table S10a. High versus low intake meta-analysis for yogurt and coronary heart disease by gender and continent. Yogurt defined as yogurt/other soured milk products. CI indicates confidence interval; RR, risk ratio.

		Number of studies	RR	95% CI	I^2 (*P* heterogeneity between studies)	*P* heterogeneity between subgroups
Yogurt	Total	3	1.04	0.95, 1.13	0% (0.42)	
	Gender					0.22
	Men	1	1.08	0.95, 1.23		
	Women	1	0.69	0.34, 1.40		
	Continent					0.26
	Asia	-	-	-		
	Europe	2	1.04	0.96, 1.13		
	North America	1	0.69	0.34, 1.40		

Supplementary Table S10b. High versus low intake meta-analysis for yogurt and ischemic stroke by gender and continent. Yogurt defined as yogurt/other soured milk products. CI indicates confidence interval; RR, risk ratio.

		Number of studies	RR	95% CI	I^2 (*P* heterogeneity between studies)	*P* heterogeneity between subgroups
Yogurt	Total	5	0.98	0.93, 1.03	42% (0.11)	
	Gender					0.72
	Men	2	1.00	0.84, 1.19		
	Women	3	0.97	0.88, 1.06		
	Continent					<0.05
	Asia	-	-	-		
	Europe	4	0.97	0.93, 1.01		
	North America	1	1.20	0.98, 1.48		

Supplementary Table S11. Linear dose-response meta-analysis for yogurt intake (per 100 g/day) and coronary heart disease by gender and continent. Yogurt defined as yogurt/other soured milk products. CI indicates confidence interval; RR, risk ratio.

		Number of studies	RR	95% CI	I^2 (*P* heterogeneity between studies)	*P* heterogeneity between subgroups
Cheese	Total	7	0.91	0.84, 0.99	37% (0.12)	
	Gender					0.03
	Men	3	1.03	0.93, 1.13		
	Women	3	0.82	0.69, 0.97		
	Continent					0.98
	Asia	-	-	-		
	Europe	6	0.91	0.84, 1.00[a]		
	North America	1	0.92	0.54, 1.58		
Low-fat cheese	Total	3	1.17	0.85, 1.61	85% (<0.01)	
	Gender					0.53
	Men	1	0.98	0.65, 1.47		
	Women	2	1.38	0.51, 3.73		
	Continent					0.33
	Asia	-	-	-		
	Europe	2	0.98	0.76, 1.26		
	North America	1	1.51	0.65, 3.52		

Supplementary Table S12a. High versus low intake meta-analysis for cheese and coronary heart disease by gender and continent. [a]*P* = 0.04 for the association between cheese and coronary heart disease in studies from Europe. CI indicates confidence interval; RR, risk ratio.

		Number of studies	RR	95% CI	I^2 (*P* heterogeneity between studies)	*P* heterogeneity between subgroups
Cheese	Total	3	0.89	0.78, 1.01	37% (0.20)	
	Gender					0.17
	Men	1	0.88	0.77, 1.01		
	Women	1	0.63	0.40, 0.99		
	Continent					0.11
	Asia	-	-	-		
	Europe	2	0.92	0.84, 1.01		
	North America	1	0.63	0.40, 0.99		

Supplementary Table S12b. High versus low intake meta-analysis for cheese and ischemic stroke by gender and continent. CI indicates confidence interval; RR, risk ratio.

		Number of studies	RR	95% CI	I^2 (*P* heterogeneity between studies)	*P* heterogeneity between subgroups
Cheese	Total	6	0.96	0.93, 0.98	3% (0.41)	
	Gender					0.55
	Men	3	0.96	0.92, 1.00[a]		
	Women	3	0.94	0.90, 0.98		
	Continent					0.90
	Asia	-	-	-		
	Europe	5	0.96	0.93, 0.98		
	North America	1	0.93	0.56, 1.53		
Low-fat cheese	Total	2	1.24	0.76, 2.04	90% (<0.01)	
	Gender					0.44
	Men	1	0.98	0.67, 1.43		
	Women	2	1.41	0.62, 3.21		
	Continent					0.27
	Asia	-	-	-		
	Europe	1	0.94	0.86, 1.02		
	North America	1	1.47	0.67, 3.21		

Supplementary Table S13a. Linear dose-response meta-analysis for cheese intake (per 20 g/day) and coronary heart disease by gender and continent. [a]$P = 0.07$ for the association between cheese and coronary heart disease in studies among men. CI indicates confidence interval; RR, risk ratio.

		Number of studies	RR	95% CI	I^2 (*P* heterogeneity between studies)	*P* heterogeneity between subgroups
Cheese	Total	3	0.96	0.91, 1.01	67% (<0.05)	
	Gender					0.14
	Men	1	0.95	0.91, 0.99		
	Women	1	0.81	0.65, 1.00[a]		
	Continent					0.09
	Asia	-	-	-		
	Europe	2	0.97	0.93, 1.01		
	North America	1	0.81	0.65, 1.00[b]		

Supplementary Table S13b. Linear dose-response meta-analysis for cheese intake (per 20 g/day) and ischemic stroke by gender and continent. [a]$P < 0.05$ for the association between cheese and ischemic stroke in studies among women; [b]$P < 0.05$ for the association between cheese and ischemic stroke in studies from North America. CI indicates confidence interval; RR, risk ratio.

		Number of studies	RR	95% CI	I^2 (*P* heterogeneity between studies)	*P* heterogeneity between subgroups
Butter	Total	4	0.99	0.92, 1.07	0% (0.86)	
	Gender					0.96
	Men	3	1.01	0.91, 1.11		
	Women	2	1.01	0.86, 1.19		
	Continent					0.98
	Asia	-	-	-		
	Europe	3	0.99	0.92, 1.07		
	North America	1	0.99	0.77, 1.27		

Supplementary Table S14. High versus low intake meta-analysis for butter and coronary heart disease by gender and continent. CI indicates confidence interval; RR, risk ratio.

		Number of studies	RR	95% CI	I^2 (*P* heterogeneity between studies)	*P* heterogeneity between subgroups
Butter	Total	3	1.00	0.99, 1.01	0% (0.85)	
	Gender					0.55
	Men	2	1.01	0.98, 1.05		
	Women	2	1.00	0.99, 1.01		
	Continent					0.58
	Asia	-	-	-		
	Europe	1	1.01	0.98, 1.05		
	North America	2	1.00	0.99, 1.01		

Supplementary Table S15. Linear dose-response meta-analysis for butter intake (per 6 g/day) and coronary heart disease by gender and continent. CI indicates confidence interval; RR, risk ratio.

Outcome	Exposure[a]	Type of meta-analysis	RR	95% CI	I^2 (*P* heterogeneity between studies)
CHD	Yogurt	High versus low	0.95	0.90, 0.99	49% (0.06)
Ischemic stroke	Milk[b]	Linear dose-response	0.97	0.94, 1.00[c]	16% (0.31)
Ischemic stroke	Cheese	High versus low	0.90	0.83, 0.99	37% (0.20)
Ischemic stroke	Cheese[d]	Linear dose-response	0.98	0.96, 1.00[e]	67% (0.05[f])
Ischemic stroke	Low-fat yogurt *for* high-fat yogurt[g]	Linear dose-response	1.25	1.03, 1.50	67% (0.08)
Ischemic stroke	High-fat yogurt *for* low-fat milk	Linear dose-response	0.83	0.72, 0.95	79% (0.03)
Ischemic stroke	High-fat yogurt *for* high-fat milk	Linear dose-response	0.81	0.69, 0.95	79% (0.03)
Ischemic stroke	Cheese *for* high-fat yogurt	Linear dose-response	1.18	1.02, 1.36	80% (0.02)

Supplementary Table S16. Meta-analysis where fixed-effects and random-effects modelling differed in conclusions. [a]Yogurt defined as yogurt/other soured milk products. [b]Per 200 g higher intake of milk/day. [c]P <0.05. [d]Per 20 g higher intake of cheese/day. [e]P = 0.04; [f]P <0.05. [g]Substitutions between dairy product subgroups (per 1 serving/day). For example in the mean of substitution of low-fat yogurt *for* high-fat yogurt; i.e. low-fat yogurt takes the place of high-fat yogurt. For milk and yogurt, the serving size was 200 g and for cheese 20 g. CHD indicates coronary heart disease; CI, confidence interval; RR, risk ratio.

Search strategy for PubMed:

Exposures:

#1: Dairy Products [MeSH] OR dairy [tiab]

Outcomes:

#2: Cardiovascular Diseases [MeSH] OR cardiovascular [tiab] OR CVD [tiab] OR "myocardial ischemia" [tiab] OR "myocardial ischaemia" [tiab] OR "ischemic heart" [tiab] OR "ischaemic heart" [tiab] OR "acute coronary syndrome" [tiab] OR "coronary artery" [tiab] OR "coronary heart" [tiab] OR CHD [tiab] OR "myocardial infarction" [tiab] OR "sudden cardiac" [tiab] OR stroke [tiab] OR cerebrovascular [tiab] OR "cerebral infarction" [tiab] OR "peripheral artery" [tiab]

Exposures and outcomes combined:

#3: #1 AND #2

The search terms were adapted for use with Embase.

Supplementary Methods. Literature search strategy.

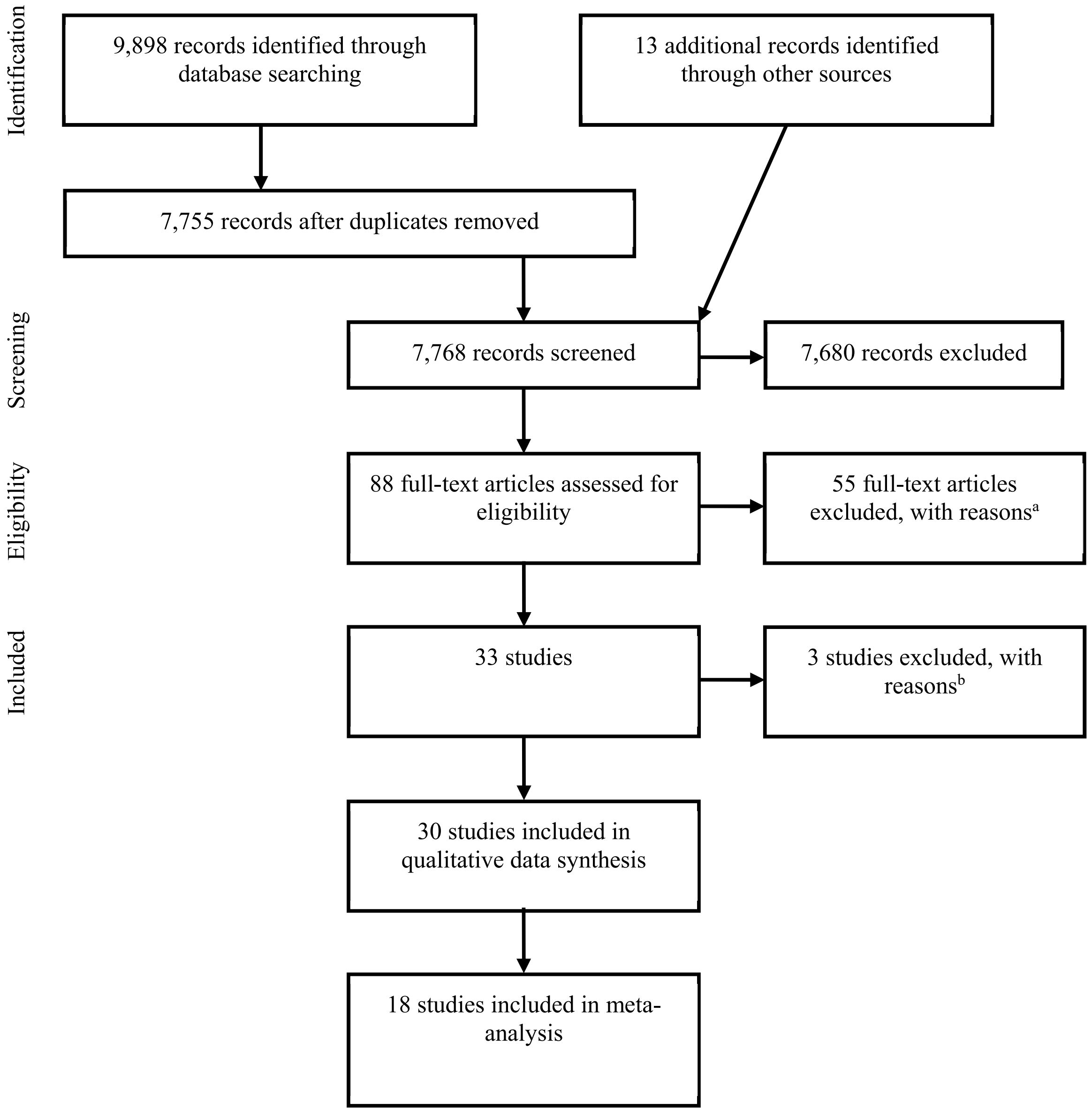

Supplementary Fig. S1. Flow diagram summarizing the study selection processes. Adapted from Liberati et al.[1]. [a]Articles excluded due to not relevant exposure (n = 35), outcome (n = 19) or study design (n = 1). [b]Studies excluded due to duplicate publications (n = 3).

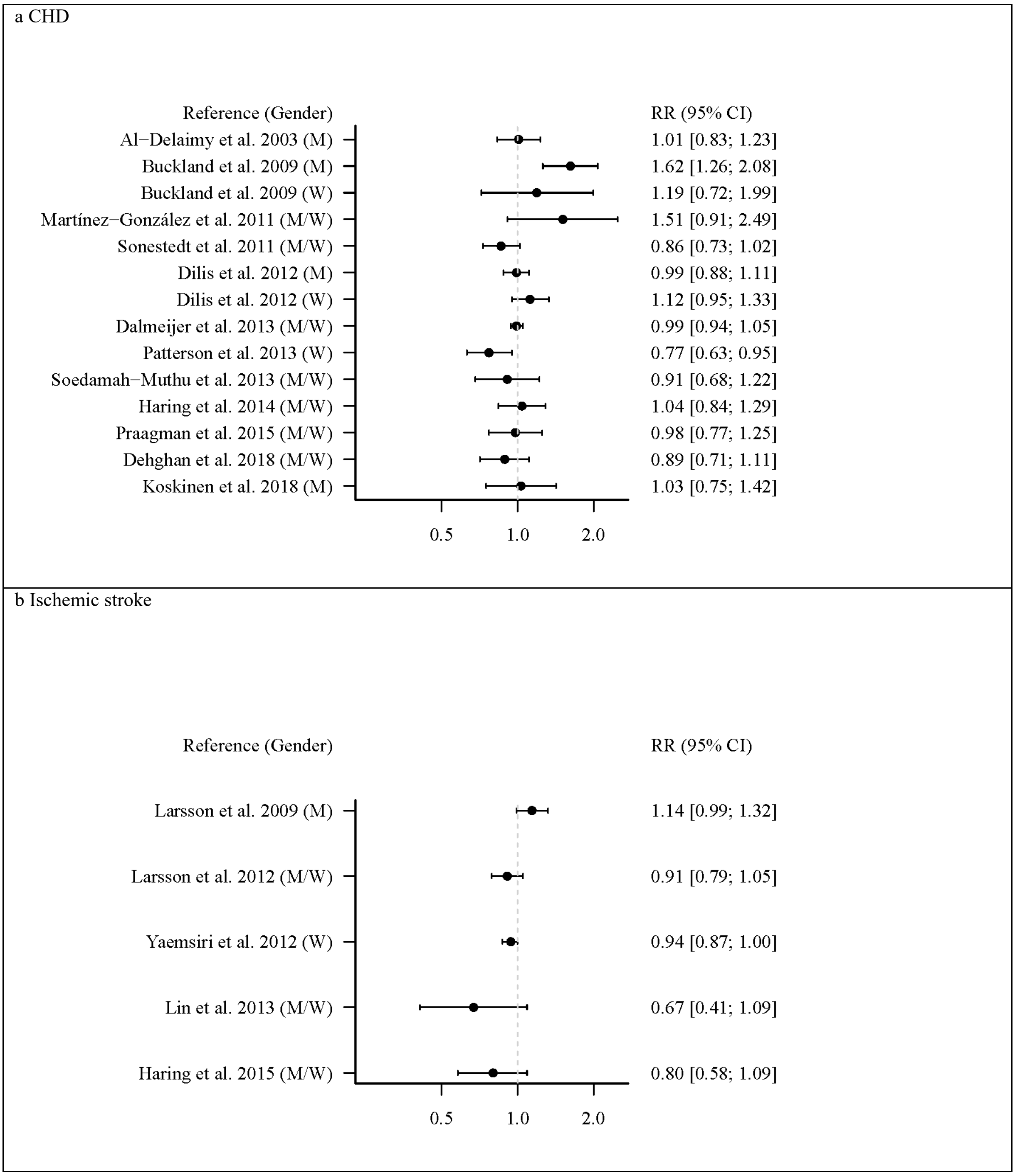

Supplementary Fig. S2. Point risk estimates of CHD (panel a) and ischemic stroke (panel b) for high versus low total intake of dairy products or trend[23–25] if high versus low intake was not reported. CHD indicates coronary heart disease; CI, confidence interval; M, males; RR, risk ratio; W, women.

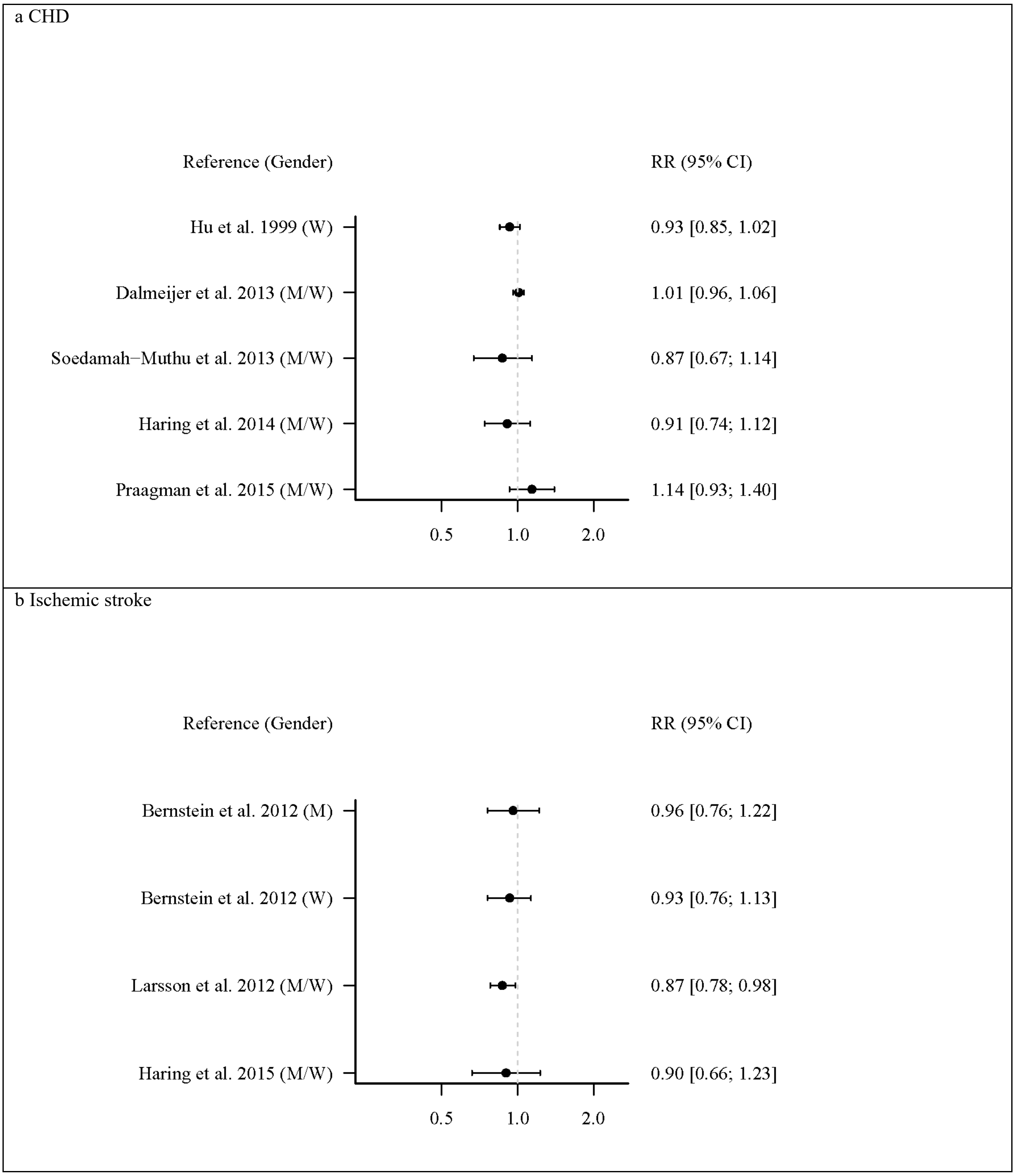

Supplementary Fig. S3. Point risk estimates of CHD (panel a) and ischemic stroke (panel b) for high versus low intake of low-fat dairy products or trend[11,24] if high versus low intake was not reported. CHD indicates coronary heart disease; CI, confidence interval; M, males; RR, risk ratio; W, women.

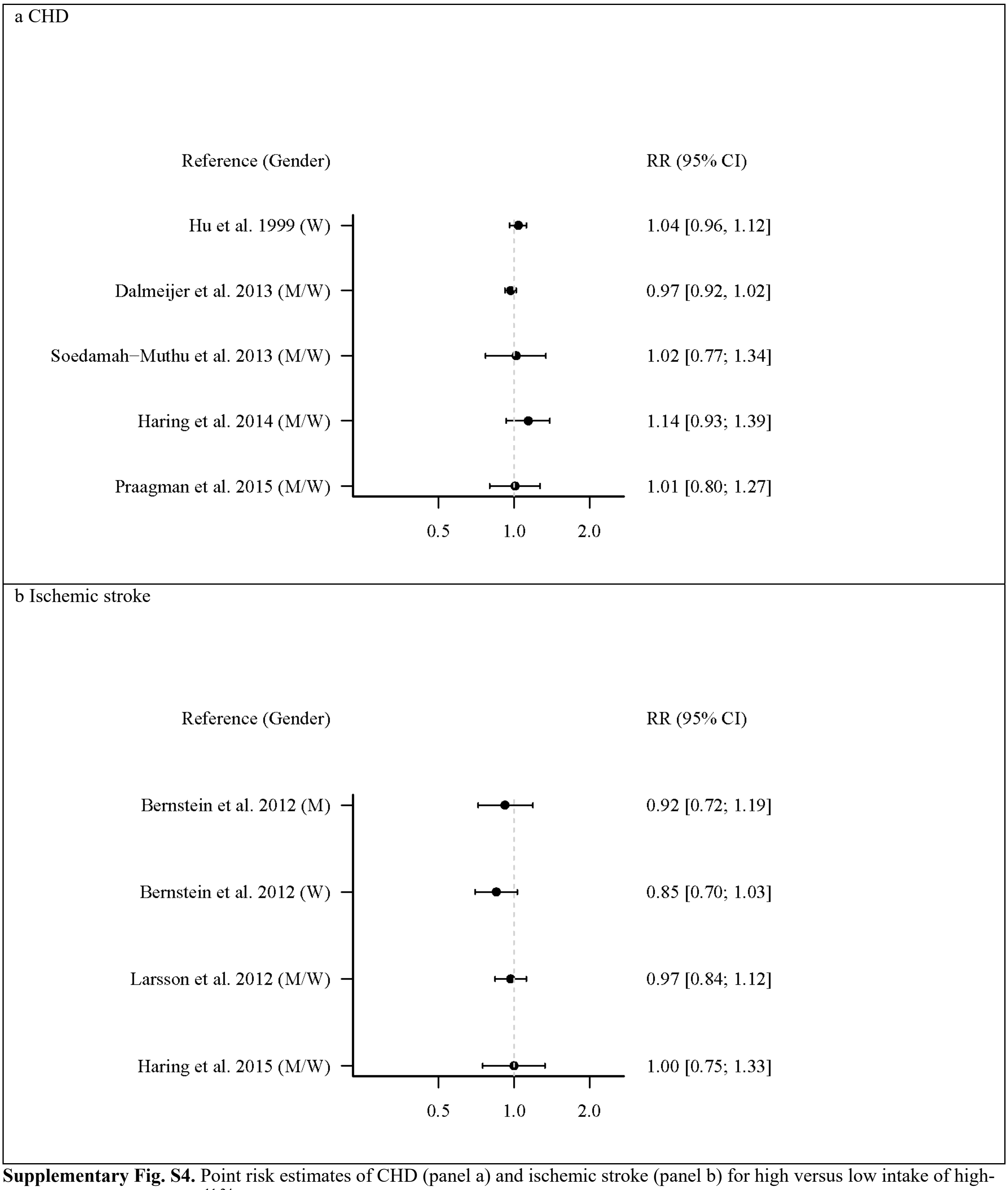

Supplementary Fig. S4. Point risk estimates of CHD (panel a) and ischemic stroke (panel b) for high versus low intake of high-fat dairy products or trend[11,24] if high versus low intake was not reported. CHD indicates coronary heart disease; CI, confidence interval; M, males; RR, risk ratio; W, women.

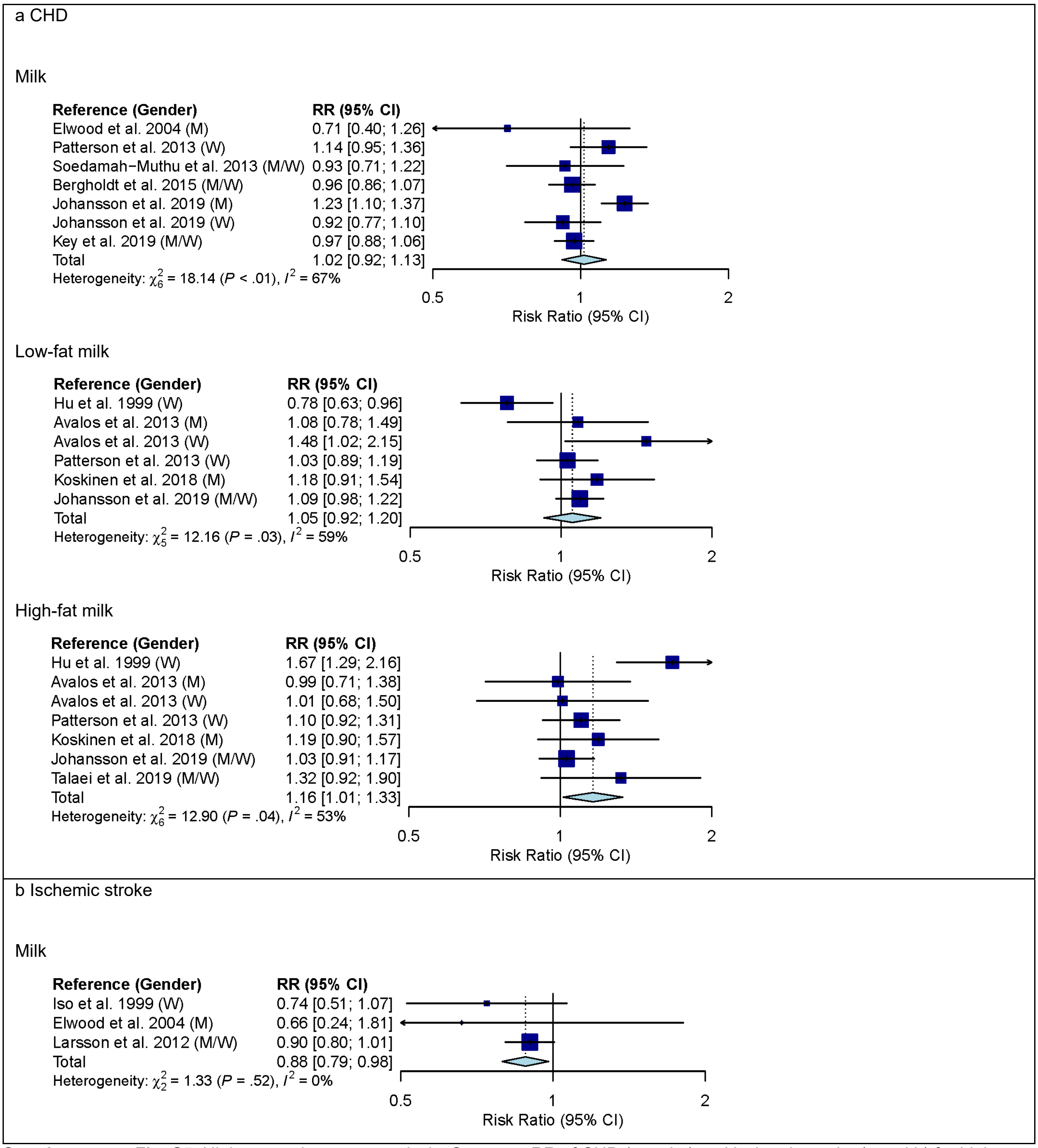

Supplementary Fig. S5. High versus low meta-analysis. Summary RR of CHD (panel a) and ischemic stroke (panel b) for high versus low intake of milk. CHD indicates coronary heart disease; CI, confidence interval; M, males; RR, risk ratio; W, women.

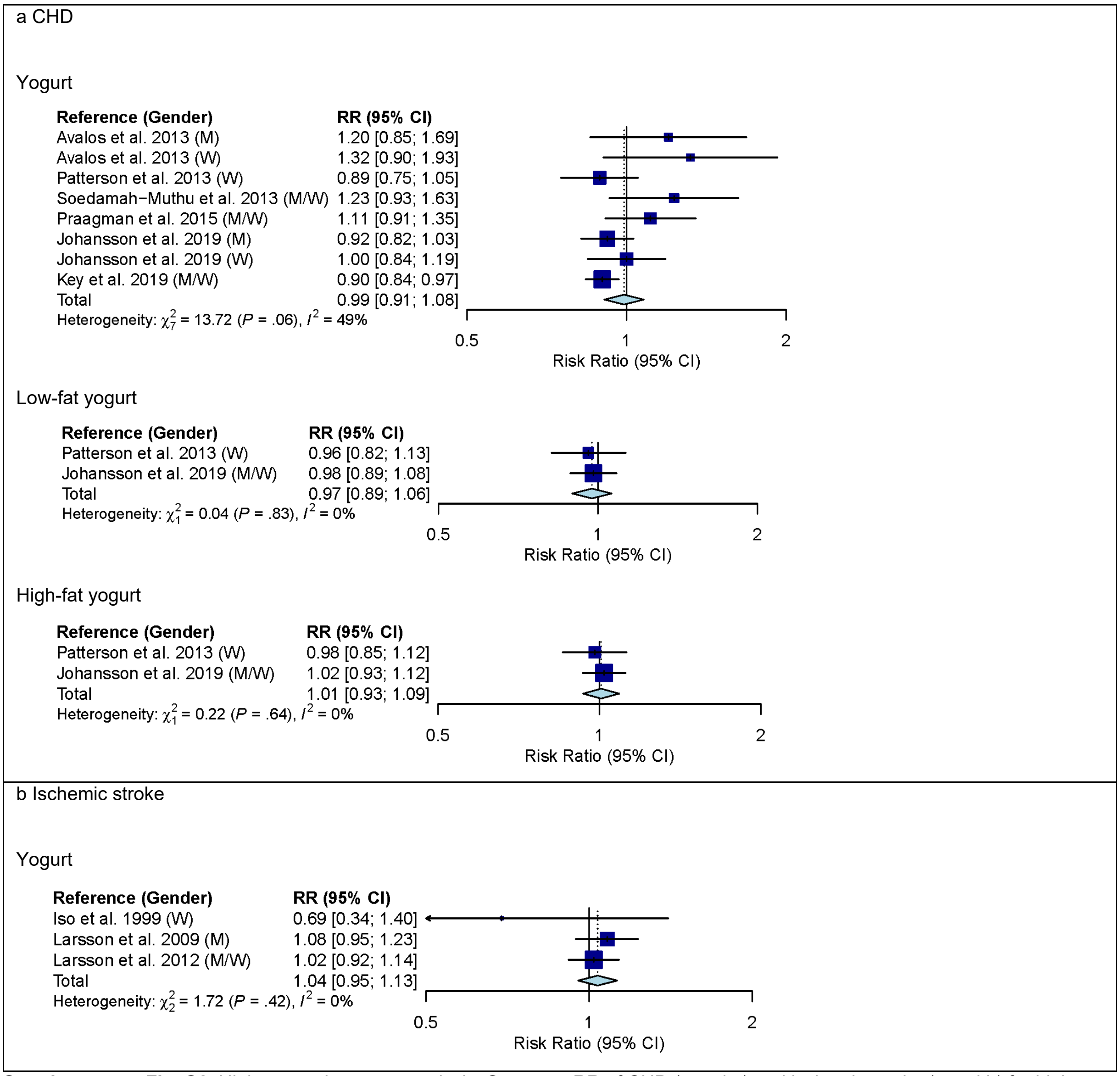

Supplementary Fig. S6. High versus low meta-analysis. Summary RR of CHD (panel a) and ischemic stroke (panel b) for high versus low intake of yogurt. Yogurt defined as yogurt/other soured milk products. CHD indicates coronary heart disease; CI, confidence interval; M, males; RR, risk ratio; W, women.

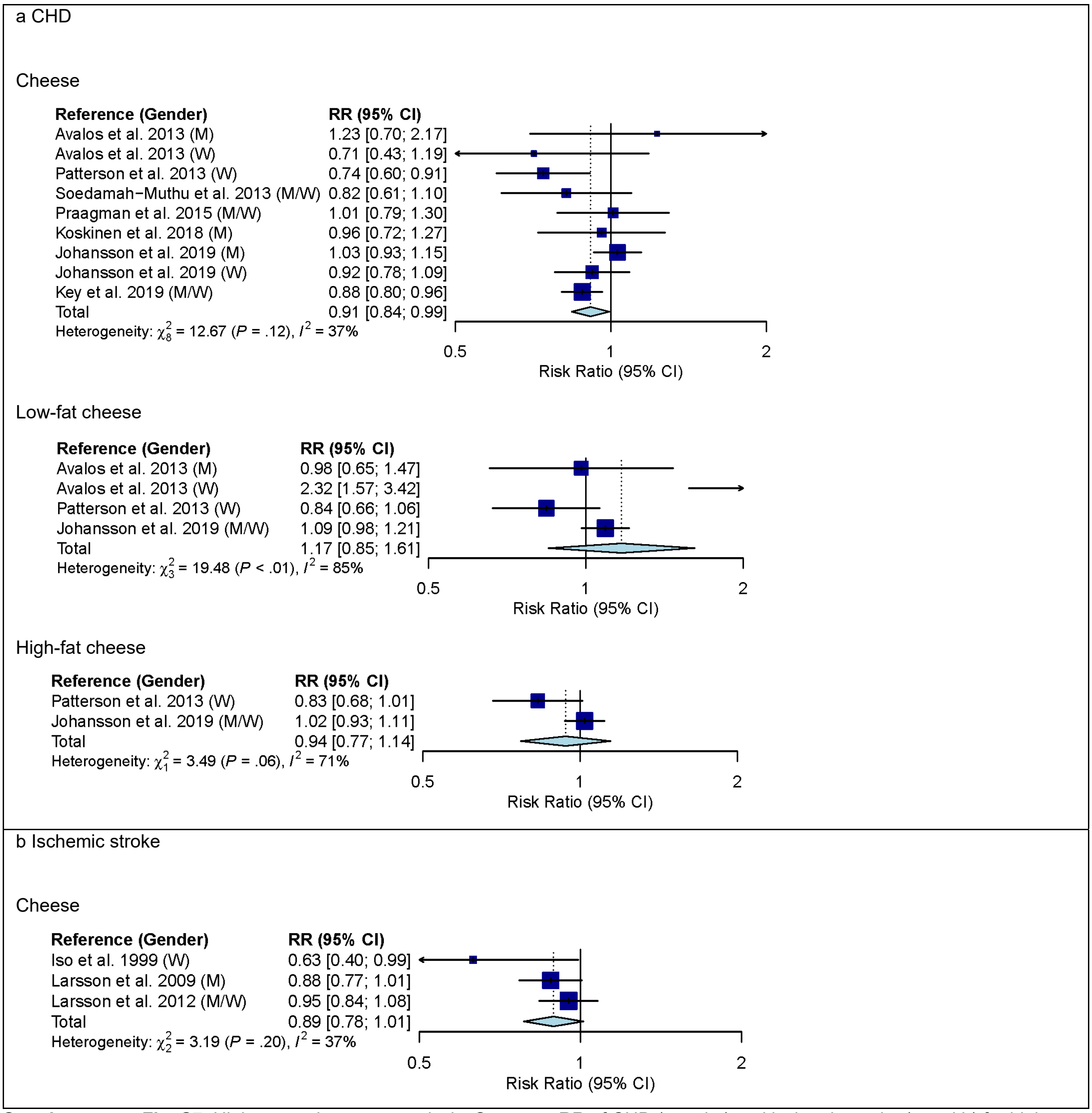

Supplementary Fig. S7. High versus low meta-analysis. Summary RR of CHD (panel a) and ischemic stroke (panel b) for high versus low intake of cheese. CHD indicates coronary heart disease; CI, confidence interval; M, males; RR, risk ratio; W, women.

Butter

Reference (Gender)	RR (95% CI)
Sonestedt et al. 2011 (M/W)	0.94 [0.81; 1.10]
Avalos et al. 2013 (M)	1.06 [0.77; 1.46]
Avalos et al. 2013 (W)	0.88 [0.58; 1.33]
Koskinen et al. 2018 (M)	1.15 [0.80; 1.65]
Johansson et al. 2019 (M)	0.99 [0.89; 1.11]
Johansson et al. 2019 (W)	1.04 [0.87; 1.24]
Total	0.99 [0.92; 1.07]

Heterogeneity: $\chi^2_5 = 1.89$ (P = .86), $I^2 = 0\%$

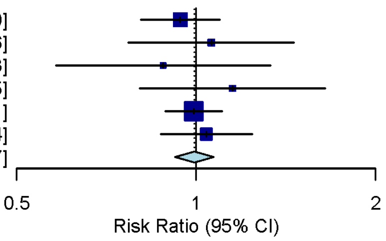

Supplementary Fig. S8. High versus low meta-analysis. Summary RR of coronary heart disease for high versus low intake of butter. CI indicates confidence interval; M, males; RR, risk ratio; W, women.

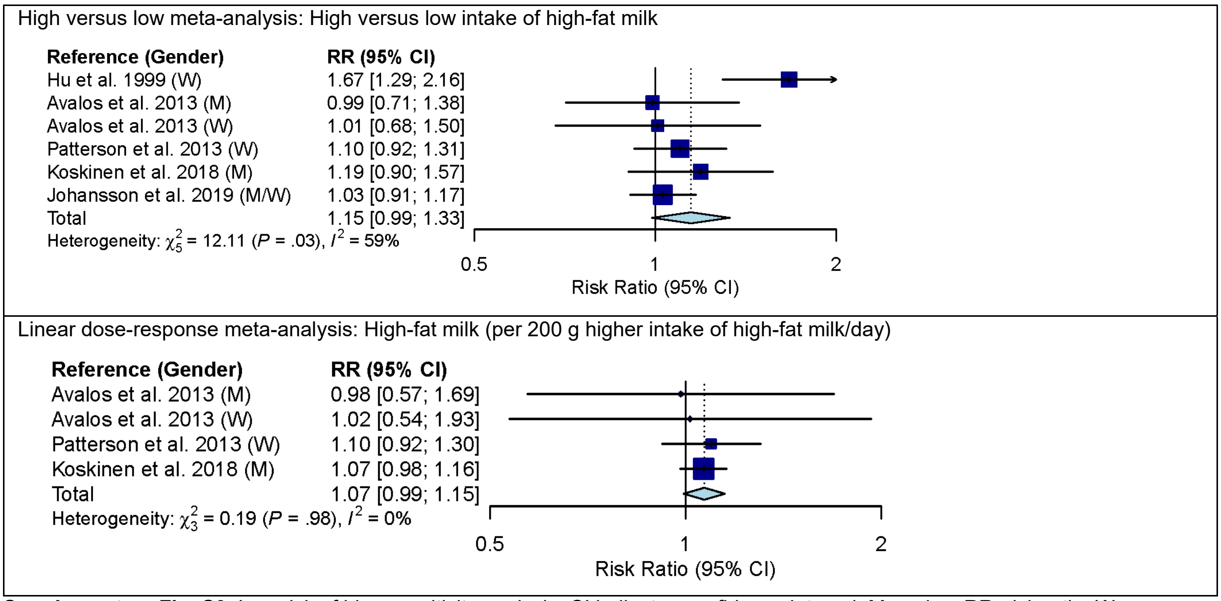

Supplementary Fig. S9. Low risk of bias sensitivity analysis. CI indicates confidence interval; M, males; RR, risk ratio; W, women.

High versus low meta-analysis: High versus low intake of yogurt

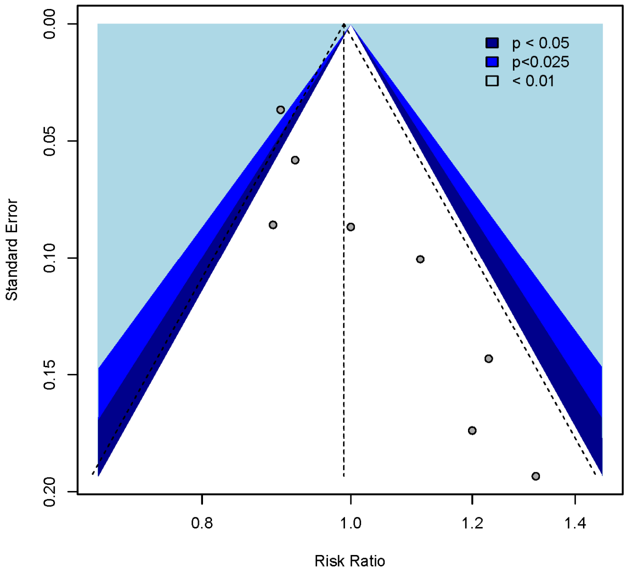

Linear dose-response meta-analysis: Yogurt (per 100 g higher intake of high-fat milk/day)

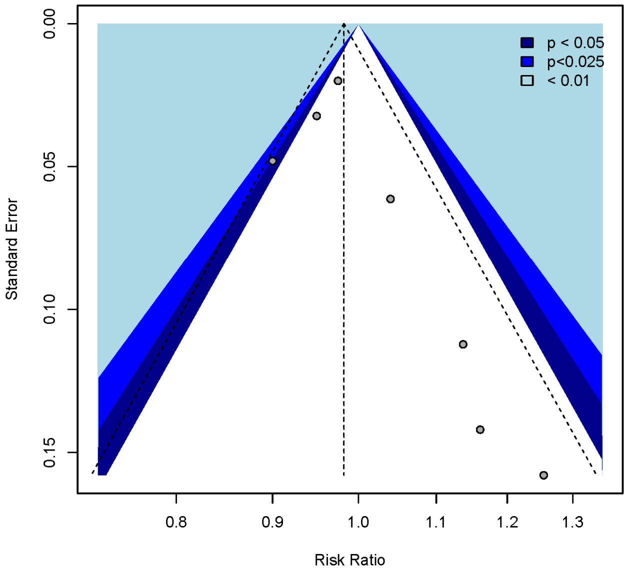

Supplementary Fig. S10. Assessment of publication bias. Funnel plots for intake of yogurt and risk of coronary heart disease. Test for publication bias: Egger's test: *P* <0.01 for high versus low meta-analysis and *P* = 0.14 for linear dose-response meta-analysis. Yogurt defined as yogurt products/other soured milk products.

References

1. Liberati, A. *et al.* The PRISMA statement for reporting systematic reviews and meta-analyses of studies that evaluate healthcare interventions: explanation and elaboration. *BMJ* **339**, b2700 (2009).
2. Ygil, K. H. Mål, vægt og portionsstørrelser på fødevarer. (In Danish). Søborg: National Food Institute, Technical University of Denmark (2013).
3. Institute of Medicine. Nutrition labeling: issues and directions for the 1990s. Washington, DC: The National Academies Press https://doi.org/10.17226/1576 (1990).
4. Wells, G. A. *et al.* The Newcastle-Ottawa Scale (NOS) for assessing the quality of observational studies in meta-analyses https://ohri.ca/programs/clinical_epidemiology/oxford.asp (2020).
5. Elwood, P. C., Pickering, J. E., Fehily, A. M., Hughes, J. & Ness, A. R. Milk drinking, ischaemic heart disease and ischaemic stroke I. Evidence from the Caerphilly cohort. *Eur. J. Clin. Nutr.* **58**, 711–717 (2004).
6. Patterson, E., Larsson, S. C., Wolk, A. & Åkesson, A. Association between dairy food consumption and risk of myocardial infarction in women differs by type of dairy food. *J. Nutr.* **143**, 74–79 (2013).
7. Soedamah-Muthu, S. S., Masset, G., Verberne, L., Geleijnse, J. M. & Brunner, E. J. Consumption of dairy products and associations with incident diabetes, CHD and mortality in the Whitehall II study. *Br. J. Nutr.* **109**, 718–726 (2013).
8. Bergholdt, H. K. M., Nordestgaard, B. G., Varbo, A. & Ellervik, C. Milk intake is not associated with ischaemic heart disease in observational or Mendelian randomization analyses in 98,529 Danish adults. *Int. J. Epidemiol.* **44**, 587–603 (2015).
9. Johansson, I. *et al.* Dairy product intake and cardiometabolic diseases in Northern Sweden: a 33-year prospective cohort study. *Nutrients* **11**, 284 (2019).
10. Key, T. J. *et al.* Consumption of meat, fish, dairy products, and eggs and risk of ischemic heart disease. *Circulation* **139**, 2835–2845 (2019).
11. Hu, F. B. *et al.* Dietary saturated fats and their food sources in relation to the risk of coronary heart disease in women. *Am. J. Clin. Nutr.* **70**, 1001–1008 (1999).
12. Avalos, E. E. *et al.* Is dairy product consumption associated with the incidence of CHD? *Public Health Nutr.* **16**, 2055–2063 (2013).
13. Koskinen, T. T. *et al.* Intake of fermented and non-fermented dairy products and risk of incident CHD: the Kuopio Ischaemic Heart Disease Risk Factor Study. *Br. J. Nutr.* **120**, 1288–1297 (2018).
14. Talaei, M. *et al.* Whole milk consumption and risk of cardiovascular disease and mortality: Isfahan Cohort Study. *Eur. J. Nutr.* **58**, 163–171 (2019).
15. Praagman, J. *et al.* Dairy products and the risk of stroke and coronary heart disease: the Rotterdam Study. *Eur. J. Nutr.* **54**, 981–990 (2015).
16. Sonestedt, E. *et al.* Dairy products and its association with incidence of cardiovascular disease: the Malmö Diet and Cancer cohort. *Eur. J. Epidemiol.* **26**, 609–618 (2011).
17. Liu, Q. *et al.* Theoretical effects of substituting butter with margarine on risk of cardiovascular disease. *Epidemiology* **28**, 145–156 (2017).
18. Iso, H. *et al.* Prospective study of calcium, potassium, and magnesium intake and risk of stroke in women. *Stroke* **30**, 1772–1779 (1999).
19. Larsson, S. C., Virtamo, J. & Wolk, A. Dairy consumption and risk of stroke in Swedish women and men. *Stroke* **43**, 1775–1780 (2012).
20. Larsson, S. C. *et al.* Dairy foods and risk of stroke. *Epidemiology* **20**, 355–360 (2009).
21. Laursen, A. S. D. *et al.* Substitutions of dairy product intake and risk of stroke: a Danish cohort study. *Eur. J. Epidemiol.* **33**, 201–212 (2018).

22. Laursen, A. S. D. *et al.* Substitutions between dairy products and risk of stroke: results from the European Investigation into Cancer and Nutrition-Netherlands (EPIC-NL) cohort. *Br. J. Nutr.* **121**, 1398–1404 (2019).

23. Dilis, V. *et al.* Mediterranean diet and CHD: the Greek European Prospective Investigation into Cancer and Nutrition cohort. *Br. J. Nutr.* **108**, 699–709 (2012).

24. Dalmeijer, G. W. *et al.* Dairy intake and coronary heart disease or stroke – A population-based cohort study. *Int. J. Cardiol.* **167**, 925–929 (2013).

25. Yaemsiri, S. *et al.* Trans fat, aspirin, and ischemic stroke in postmenopausal women. *Ann. Neurol.* **72**, 704–715 (2012).
